# The impact of COVID-19 on music consumption and music spending

**DOI:** 10.1371/journal.pone.0267640

**Published:** 2022-05-13

**Authors:** Janis Denk, Alexa Burmester, Michael Kandziora, Michel Clement

**Affiliations:** 1 Institute for Marketing, Hamburg Business School, University of Hamburg, Hamburg, Germany; 2 Department of Leadership and Management, Kühne Logistics University, Hamburg, Germany; Iowa State University, UNITED STATES

## Abstract

COVID-19 induced restrictions ordered by governments around the world have been an exogenous shock to the music industry, which we divide into two affected groups: 1) live music events and 2) recorded music. While the impact on live music events is rather obvious, it is unclear how the current pandemic is affecting the recorded music market. Hence, we study consumers’ pre- and post-pandemic shifts in consumer spending (in euros) and music consumption (in hours) across live music events, as well as the digital and physical submarkets of recorded music, in the world’s fourth largest music market, Germany. Relying on an online bi-annual panel capturing five waves between winter 2018/19 and winter 2020/21, we find that the COVID-19 pandemic is accelerating the continuous trend towards digitalization of the music landscape with premium streaming being the biggest beneficiary. However, total monthly consumer spending on music decreased by more than 45% compared to pre-pandemic, with live music events and physical sales being the most severely affected. Surprisingly, music consumption in hours also decreased during the lockdown even though consumers spent more time at home.

## Introduction

The COVID-19 outbreak has substantially impacted the global economy [1]. While some industries have benefited from the pandemic (e.g., pharma and cybersecurity), others have substantially suffered (e.g., airlines and hotels). The restrictions ordered by governments around the world also resulted in an exogenous shock to cultural markets–especially for live events, but also for all other channels–in accessing and consuming music.

This research focuses on the impact of COVID-19 on the music industry across the live music events market, as well as digital and physical submarkets of recorded music. Specifically, we study consumers’ pre- and post-pandemic shifts in consumer spending (in euros) and music consumption (in hours). Pre-pandemic predictions by PwC [2] for the German music market (the world’s fourth-largest market in terms of revenue; [3]) forecast a stable average growth rate of 2.8% in sales volume between 2019–2023. This prediction has been challenged due to the COVID-19 outbreak. Specifically, post-pandemic effects remain unclear and may differ across the different submarkets. On the one hand, digital purchase channels, particularly music streaming, potentially benefit from a pandemic as people can spend more time consuming music at home during lockdowns. On the other hand, severe restrictions in public life, especially closed stores or cancelled live music events, in addition to the general economic downturn, reduce demand. Therefore, the overall impact of COVID-19 on the music industry is uncertain as the submarkets are impacted differently.

Considering these opposing effects, our study estimates the total impact of the exogenous shock at the individual level, and focuses on (1) *consumer spending* and (2) *music consumption* across various submarkets. As the uncertainty resulting from the pandemic affects consumers directly, we rely on a consumer-centric analysis of the German music market. Our data consists of an ongoing online panel that was set up with 3,365 participants selected to represent the German population in winter 2018/19 [4]. Since then, we have conducted our online survey every six months resulting in five waves of panel data. The last wave was collected in winter 2020/21. For our analysis, we considered only those respondents who participated throughout all five surveys, leading to a data set with 594 respondents. The timing of the data collection enables us to analyze pre- and post-COVID-19 behavior across the bi-annual waves. We also collected data from the following areas: demographics, consumer spending, and music consumption, to control for industry effects and heterogeneity.

We find that COVID-19 is accelerating the continuous trend towards digitalization of the music landscape with premium streaming being the biggest beneficiary. However, total monthly *consumer spending* on music has decreased by more than 45% compared to pre-pandemic, with live music events and physical sales most severely affected. In spite of the COVID-19 outbreak and the fundamental change from analog to digital music, spending on digital downloads has also decreased. This suggests an expiring revenue stream in the mid-run. Surprisingly, the pandemic is also accelerating the ongoing trend of decreasing *music consumption* in hours even though consumers spend more time at home.

Our results contribute to academic literature in many ways. While Carlson et al. [5] provide initial insights on the impact of COVID-19 on music consumption, we expand their findings by using a panel model which allows us to analyze consumer behavior before and after the pandemic outbreak across different channels more precisely. In addition, we also analyze the impact of COVID-19 on consumer spending. Consequently, our results provide managerial implications for labels, artists, platforms, ticketers, and event organizers. Moreover, our results might trigger discussions about new types of contracts between artists, labels, and platforms, accounting for the unique risk-reward attribute of live music events which has now become apparent during the pandemic.

Next, we describe the pre-pandemic market situation for the German market and outline our expectations with respect to post-pandemic effects across the subindustries. After a short literature overview, we present our model and data, and then discuss our results. Finally, we provide implications for managers and researchers.

## Evolution of the music industry–Recorded music and live music markets

In this section, we describe the music industry in Germany before and after the COVID-19 outbreak.

### Pre-COVID-19 outbreak

In terms of revenue, the music industry is the most important entertainment industry in Germany, even exceeding both the book (excluding textbooks) and game markets. In 2017, sales volume was inflation-adjusted (based on prices in 1995) valued at 3.75 bn EUR [6]. When it comes to its products, the music industry can be divided into two categories: live music and recorded music [7].

While live music events only contributed 48% of total revenues (2.45 bn EUR) in the 1995 German music market, it contributed as much as 72% of total revenues (inflation adjusted 2.69 bn EUR) in 2017 [6]. This is in line with Black, Fox, and Kochanowski [8] who argue that touring can generate 75% of any artist’s income and thus “pays the bills for most artists.” While Montoro-Pons and Cuadrado-García [7] see a remarkable growth, especially in the demand for summer music festivals, the Federal Association of the Events Industry [6] argues that the recent revenue growth can be attributed to rising ticket prices as visitor numbers are currently declining.

On the contrary, the absolute amount of revenues from recorded music significantly decreased from 2.65 bn EUR in 1995 to an inflation adjusted 1.06 bn EUR in 2017 [6]. Hence, the growing revenue share of live music events is not only due to increasing live music revenues, but is also due to decreasing revenues from recorded music. Revenues from recorded music can be divided into revenues from physical, digital downloads, and streaming. While the market for recorded music reached its peak in 1997, the digital disruption in the early 2000s led to a slump in revenues that continued up until 2013 [9]. This development was mostly due to a substantial drop in physical sales which could not be offset by relatively low revenues from paid downloads. However, while physical sales are still decreasing and revenues from paid downloads start to decrease, the market observes a renewed growth in revenues from recorded music driven by music streaming from 2013 onwards. The streaming market allocates the revenues across right holders dependent on usage–thus, the listening time is a highly relevant indicator for the allocation mechanism.

The listening time across all channels has been rather stable over the last years [9]. The picture is different when comparing music purchase shares and music listening behavior. In Germany live music only accounts for 1.4% and recorded music for 98.6% of consumers’ total listening time [9]. The traditional radio clearly dominates in the recorded music segment, although the share of the total listening time decreased from 42.1% in 2013 to 27% in 2017 [10,11]. However, the share of online radio slightly increased in the same period from 8.8% to 10.3%. In analogy to the development in revenues, it is possible to see a substantial increase in listening time from premium streaming (from 2.4% to 7.4%) and free streaming (from 3.2% to 8.1%), while the listening time of physical sound carriers and files from digital downloads has decreased.

To summarize, in pre-COVID-19 times, both the market for recorded music and the market for live music showed a steady revenue growth. In the case of the recorded music market, the growth was driven by music streaming, while higher ticket prices increased live music revenues. Consequently, PwC predicted an average growth rate of 2.8% for the German music market between 2019–23 before the COVID-19 outbreak [2].

### Post-COVID-19 outbreak

The spread of COVID-19 in Germany and the associated restrictions represent a structural break in the period of increasing revenues within the music industry. The outbreak of COVID-19 has led to an economic downturn and job insecurity [12] which adversely impact total consumer expenditures for hedonistic products including musical goods. The pandemic created frictions on the supply side [13] resulting in less music production and releases [14,15]. A reduction in new music (supply) may also fundamentally reduce consumers’ total consumption and spending (demand) as music fans put a high value on listening to the latest songs [16]. Moreover, certain restrictions during the pandemic, such as closed stores [14], directly affect physical music sales, while physical music consumption might not be affected as strongly because consumers can still listen to their (favorite) records. The ban on large-scale events directly and substantially affects consumers’ live music spending and listening behavior [14]. In addition, fewer consumption situations, for example a decrease in commuting time or closed gyms, might also affect the consumption time of recorded music [17,18]. A decrease in commuting time is especially likely to have a negative impact on radio music consumption. Radio music spending is not affected due to mandatory broadcasting fees in Germany. On the contrary, home office ordinances and governmental restrictions during the pandemic might increase online radio usage as radio is still a medium that keeps society connected [19].

Beyond these salient factors there are socio-cultural factors with unclear effects on the music industry. Consumers perceive music as a daily tool to cope with this pandemic [20]. However, it is unclear if escaping from society, focusing on oneself, and potentially more time for in-home consumption [17] leads to more recorded music consumption and music spending or distraction by other means like watching television or reading the news [18,21].

Potential pandemic beneficiaries are digital channels [12], such as online radio services or premium and free streaming services, which do not necessarily require physical social interactions. Premium streaming consumption and spending might particularly benefit as this channel is largely based on robust subscription models with low monthly payments [18].

Based on expected consumer behavior within the music industry during COVID-19, Table 1 summarizes our propositions of the COVID-19 impact on music spending and music consumption across different music channels.

**Table 1:** Propositions based on COVID-19 news reporting on music spending and consumption.

*Music channel by type*	*COVID-19 impact*	
	*Music spending* *(in euros)*	*Music consumption* *(in hours)*
**Total Music Market**	–	+ / –
** Live Music**	–	–
** Recorded Music**	+ / –	+ / –
Premium Streaming	+	+
Free Streaming	n/a	+
Physical	–	o
Digital Downloads	+	+
Radio	o	–
Online Radio	+	+

Note: + = positive; o = neutral;– = negative; n/a = not applicable.

## Literature overview

Table 2 gives an overview of the various related streams of empirical literature and the contribution of this study. We identify three main streams: the first investigates consumer behavior based on data *before the outbreak* of COVID-19 (e.g., [7,22–28]), the second examines consumer behavior relying on data *during the pandemic* (e.g., [5,20,29–32]), and the third stream provides evidence on the impact of COVID-19 comparing effects *before and during the outbreak* on consumer behavior in the music industry (e.g., [33–35]).

**Table 2:** Literature overview on music consumption and music spending.

Literature stream	Author/s (year)	Music industry					Dependent variables		COVID-19		Data		
		*Recorded* ^*1*^				*Live*	*Consumption*	*Spending*	*Pre-pandemic*	*Pandemic*	*Primary data* ^*2*^		*Secondary data* ^*3*^
		*Streaming*	*Physical*	*Digital*	*Radio*						*Single* *period*	*Multiple periods*	
(1) Pre-pandemic data	Montoro-Pons & Cuadrado-García (2011)		x	x	x	x	x		x		x		
	Magaudda (2011)		x	x			x		x		x		
	Papies, Eggers & Wlömert (2011)	x						x	x		x		
	Handke (2012)			x			x		x			x	
	Nguyen, Dejean & Moreau (2014)	x	x			x	x		x		x		
	Aguiar & Martens (2016)	x	x	x			x		x			x	
	Datta, Knox & Bronnenberg (2017)	x		x			x		x			x	
	Bello & Garcia (2021)	x		x			x		x				Spotify, Apple
(2) Pandemic data	Carlson et. al (2021)	x	x		x	x	x			x	x		
	Cabedo-Mas, Arriaga-Sanz & Moliner-Miravet (2021)	n/a					x			x	x		
	Fink et. al. (2021)	n/a					x			x	x		
	Hurwitz & Krumhansl (2021)	x			x		x			x	x		
	Ziv & Hollander-Shabtai (2021)	n/a					x			x	x		
	Vandenberg, Berghman & Schaap (2021)					x	x			x			Facebook
(3) Pre- and pandemic data	Yeung (2020)	x					x		x	x			Spotify
	Sim et. al (2021)	x					x		x	x			Spotify
	Váradi (2021)					x	x		x	x		x	
	**Denk, Burmester, Kandziora, Clement (2022)**	**x**	**x**	**x**	**x**	**x**	**x**	**x**	**x**	**x**		**x**	

Note

1) n/a = no information about subindustry specified

2) Primary data is categorized by single data collection and multiple data collection points

3) Secondary data based on company data.

The first stream reflects evidence on increasing digital and streaming music consumption and shifts between markets *before the outbreak* of COVID-19 [e.g.,^7^,^22^–^28^]. However, these studies do not compare consumption and spending behavior before and after the outbreak of COVID-19.

The second literature stream provides evidence on music consumption behavior *during the pandemic*. These studies investigate the impact of COVID-19 on music consumption habits [e.g.,^5^,^20^,^29^–^32^] based on data collection during the pandemic. For example, Carlson et al. [5] investigate the role of music from a psychological perspective (e.g., for mood regulation) and Vandeberg et al. [32] reveal evidence of an increase in live streaming concerts during the COVID-19 imposed lockdown in Europe. However, as their data collection is based on single periods and was not continuously collected at different times before and after the pandemic outbreak, it is unknown to what extent results are generalizable to explain music consumption behavior, making further research necessary. Beyond that, none of these studies evaluate the impact on consumers’ spending behavior nor simultaneously consider consumption effects on both recorded and live music markets.

The third literature stream shows empirical evidence on shifts in music consumption and spending behavior by comparing behavior *before and during the outbreak of COVID-19*. Using data from Spotify, Yeung [33] and Slim et al. [34] investigate the impact of COVID-19 on music streaming consumption, while Váradi [35] addresses the pandemic impact on classical concerts in the live market. Again, however, these studies do not consider consumer spending behavior. Existing music literature discusses the impact of the outbreak solely focused on single markets (e.g., streaming) but does not consider the interrelatedness of the recorded and live market. We address this research gap by analyzing consumer behavior before and after the outbreak using a bi-annual panel capturing five waves between winter 2018/19 and winter 2020/21. This extensive panel data enables us to estimate the impact of COVID-19 across various channels in the recorded market (streaming, physical, digital, and radio) and live market on music consumption and spending.

Next, we describe the model that we use to test our propositions and estimate the magnitude of the impact of COVID-19 on music consumption and spending.

## Model

As we are interested in the impact of COVID-19 on individuals’ (a) spending and (b) consumption behavior, we model the total consumer spending and the total consumption separately. For both, consumer spending and music consumption, we use a log-linear model due to the distribution of the dependent variables. We estimate the parameters using a fixed effects panel estimator with robust standard errors.

We evaluate the different impacts of COVID-19 on the two main submarkets (recorded and live) for music spending and consumption by modeling the spending (consumption) of live music and recorded music separately and following the same panel structure.

We estimate three equations for consumer spending, one for each of the different market levels *l*: total, live, and recorded, where *total* covers all spending across all channels, *live* focuses on the spending on live music, and *recorded* refers to the spending on all channels of recorded music (e.g., premium streaming, CDs). The model for consumer spending is presented in Eq 1:

$${ln\_ConsumerSpending}_{lit}=x_{it}\beta_{l}+v$$ [1]

Where *ln_ConsumerSpending*_*lit*_ = ln(*ConsumerSpending*_*lit*_ + 1), ***l*** denotes the respective market level, ***i*** the respective participant, and ***t*** time of the survey, ***β***_***l***_ is the vector of coefficients corresponding to the independent variables ***x***_***it***_. The vector ***x***_***it***_ includes a post-COVID-19 dummy variable to measure the post-COVID-19 effect, a summer dummy variable to capture seasonal effects, and relevant control variables (music education, music appreciation, active listening, preference for mainstream music, purchase reasons, marital status, education level, occupation, age, number of children, and the natural log of income).

Comparable to the model structure of consumer spending, we model music consumption for the three market levels *l* as shown in Eq 2:

$${ln\_MusicConsumption}_{lit}=x_{it}\gamma_{l}+v$$ [2]

Along with the post-COVID-19 dummy variable measuring the post-COVID-19 effect, the vector ***x***_***it***_ includes all control variables of the consumer spending model except for the purchase reasons as these are not relevant to the consumption of music.

We further analyze a share model for both consumer spending and music consumption as we are not only interested in the main effect of the COVID-19 outbreak on the general amount of consumer spending and music consumption, but also in the shifts between the different channels within the recorded market. As the budget for music spending (time for consumption) is limited and the spending on different music products from the different channels are related, we model this dependency by using the shares of the respective channel relative to the overall spending on recorded music products. We use a fractional multinomial logit model to estimate the share models that we estimate by quasi-maximum likelihood with cluster-robust standard errors with respect to participant**s** [36].

The share model for consumer spending is:

$${s\_ConsumerSpending}_{kit}=\frac{\exp\left(x_{it}\delta_{k}\right)}{\sum_{m=1}^{M}\exp\left(x_{it}\delta_{m}\right)},k=1,\ldots ,M$$ [3]

Where *s*_*ConsumerSpending*_*kit*_ denotes the share of *k*’s channel for participant *i* at time *t* for consumer spending. The channels (*k*) are premium streaming, physical (e.g., CDs, DVDs, LPs), and digital downloads. We further include a share variable for no spending which is 0 if the participant spent money at least on one channel for music at time *t* and which is 1 in the case **of** no money on recorded music spent at time *t* by participant *i*. In our case, the fractional multinomial logit model is appropriate as it allows extremes of the shares (e.g., *s* = 1 or *s* = 0) with nonzero probability [37]. ***δ***_*k*_ is the vector of coefficients corresponding to the independent variables ***x***_***it***_. The vector ***x***_***it***_ includes the post-COVID-19 dummy variable to measure the post-COVID-19 effect and the specified set of control variables of Eq 1, as well as the gender dummy.

Since all M of the ***δ***_*k*_ will not be separately identified in the multinomial quasi-likelihood, some normalization is required [37]. Thus, ***δ***_*M*_ = 0 is used, giving:

$${s\_ConsumerSpending}_{kit}=\frac{\exp\left(x_{it}\delta_{k}\right)}{1+\sum_{m=1}^{M-1}\exp\left(x_{it}\delta_{m}\right)},k=1,\ldots ,M-1$$ [4]

and

$${s\_ConsumerSpending}_{Mit}=\frac{1}{1+\sum_{m=1}^{M-1}\exp\left(x_{it}\delta_{m}\right)}.$$ [5]

Due to the normalization, we report the average partial effects (APEs) of ***δ***_*k*_ [37]. We analogously model the share model for consumers’ music consumption of recorded music in which the dependent variables are the shares for the following channels: premium streaming, free streaming, digital downloads, physical, radio, and online radio, and include the specified set of control variables of Eq 2, as well as the gender dummy.

## Research design

### Data

The Dean of Research from the Business School of the University of Hamburg reviewed and approved this research proposal with respect to ethics before the data was collected in June 2018. All surveys were conducted via Respondi–a professional market research company (https://www.respondi.com/EN/). The company provides an online access panel. Respondents need to agree to participate in surveys with Respondi (participant consent). Each survey is reviewed by the company before it is distributed online to ensure consent. Thus, the market research firm manages participant consent. The data used for this analysis is based on the “Musikstudie” [4]. The data collection was financed by the City of Hamburg and all major players in the German music industry. We use this data to study the COVID-19 effect in an independent way. This means that this study has not been financed by the partners of the “Musikstudie”.

Our empirical study relies on extensive panel data at the individual level in the areas of music consumption, consumer spending, and control variables, including demographics that were collected through five online surveys starting with 3,365 respondents in winter 2018/19 [4]. This study was designed to analyze and predict future consumer music discovery, consumption, and spending behavior in Germany. We report further information on the online survey in Table S4 in S1 File.

The panel structure of the data allows us to analyze pre- and post-COVID-19 behavior across five bi-annual waves of which three were collected before the COVID-19 outbreak (winter 2018/19, summer 2019, winter 2019/20) and two after the outbreak (summer 2020, winter 2020/21). For our first wave in winter 2018/19, we observe that our full sample is representative for the German target population ([38]; Table S5 in S1 File). Comparing the age and gender distribution of all participants of the first wave with a subsample of participants who completed all five surveys, we observe a stronger panel attrition for females and younger participants. But as we are interested in the COVID-19 effect, we decided to balance our panel and focus on participants who completed all five surveys to ensure variance in the post-COVID-19 variable for all participants. The balanced panel includes 594 respondents between the ages of 17 and 70 from the first to the last wave. We conducted two robustness checks to ensure that panel attrition has no effect on our results. First, we weighted our panel observations with respect to age and gender proportions and estimated a weighted fixed effects panel model. The estimated models remain stable (Tables S6-S9 in S1 File). Second, we estimated the models based on the unbalanced panel. Once again, the results remain robust (Tables S6-S9 in S1 File).

### Dependent variables

In order to quantify the impact of COVID-19 on (a) consumer spending and (b) music consumption, we rely on the dependent variables that are depicted in Table 3. Table 3 reports the mean values (standard deviations) of the respective variables across the panel waves.

**Table 3:** Dependent variables.

*Variable*	*Description*	*Type*	*Pre-COVID outbreak*						*Post-COVID outbreak*							
			*Winter* *2018/19*		*Summer* *2019*		*Winter 2019/20*		*Summer* *2020*		*Winter 2020/21*		*Total (all waves)*			
			*Mean*	*SD*	*Mean*	*SD*	*Mean*	*SD*	*Mean*	*SD*	*Mean*	*SD*	*Mean*	*SD*	*Min*	*Max*
**Spending_Total**	**Total consumer spending on music (in €) as a sum of:**	**Continuous**	31.99	89.17	35.70	71.97	31.45	72.97	14.59	38.65	13.63	37.51	25.47	65.98	0.00	1550.00
**Spending_Live**	**Consumer spending on live music (in €)**	**Continuous**	14.20	45.78	21.17	58.96	14.58	44.64	3.26	21.49	0.30	3.46	10.70	40.81	0.00	600.00
**Spending_Recorded**	**Consumer spending on recorded music (in €) as sum of:**	**Continuous**	17.80	70.45	14.53	36.05	16.87	53.03	11.33	31.32	13.32	37.15	14.77	47.85	0.00	1450.00
Spending_Stream	Consumer spending on streaming music (in €)	Continuous	2.34	7.54	2.21	6.32	2.73	9.11	2.32	6.55	3.14	10.69	2.55	8.21	0.00	150.00
Spending_Physical	Consumer spending on physical music (in €)	Continuous	13.17	66.36	9.59	30.54	10.87	40.13	6.37	21.52	8.06	31.74	9.61	41.07	0.00	1400.00
Spending_Digital	Consumer spending on digital music (in €)	Continuous	2.28	12.16	2.73	16.63	3.27	29.89	2.64	20.60	2.12	12.79	2.61	19.51	0.00	700.00
**Consumption_Total**	**Total music consumption (in hours) as a sum of:**	**Continuous**	22.17	20.95	21.10	20.48	20.36	19.56	19.29	19.78	19.00	19.31	20.38	20.04	0.00	160.00
**Consumption_Live**	**Consumption of live music (in hours)**	**Continuous**	0.45	1.94	0.57	2.12	0.32	1.39	0.24	2.33	0.02	0.30	0.32	1.78	0.00	40.00
**Consumption_Recorded**	**Consumption of recorded music (in hours) as sum of:**	**Continuous**	21.72	20.81	20.53	20.09	20.04	19.42	19.05	19.45	18.98	19.28	20.06	19.83	0.00	160.00
Consumption_Premium_Stream	Consumption of premium streaming music (in hours)	Continuous	1.67	5.55	2.05	7.98	2.03	5.92	2.07	5.64	2.24	6.21	2.01	6.32	0.00	100.00
Consumption_Free_Stream	Consumption of free streaming music (in hours)	Continuous	0.81	2.43	0.76	2.80	0.86	4.67	0.68	2.48	0.59	2.25	0.74	3.06	0.00	94.00
Consumption_Digital	Consumption of digital downloads (in hours)	Continuous	3.23	6.82	2.86	6.58	2.68	5.78	2.41	5.21	2.66	6.74	2.77	6.26	0.00	80.00
Consumption_Physical	Consumption of physical music (in hours)	Continuous	2.54	5.70	2.55	6.63	2.11	4.79	2.09	5.85	1.97	4.54	2.25	5.55	0.00	100.00
Consumption_Radio	Consumption of radio music (in hours)	Continuous	10.45	14.80	9.51	12.30	9.41	12.69	8.72	12.85	8.05	12.27	9.23	13.03	0.00	150.00
Consumption_Online_Radio	Consumption of online radio music (in hours)	Continuous	3.02	7.10	2.81	7.10	2.94	7.04	3.08	8.76	3.46	9.60	3.06	7.99	0.00	120.00

Note: N = 2970 observations (594 respondents) for consumer spending N = 2960 observations (592 respondents) for music consumption; consumer spending refers to the amount spent on music during the last 30 days; music consumption refers to the hours listened to music during the last seven days; the grey shaded area marks the post-COVID-19 time. Exact bi-annual data collection dates for our five periods are as follows: winter 2018/19 in January/February 2019, summer 2019 in July 2019, winter 2019/20 in January 2020, summer 2020 in June 2020, and winter 2020/21 in January 2021.

In addition to the variables capturing the total consumer spending over the last 30 days and the total music consumption over the last seven days, we also consider the subcategories of consumer spending and music consumption that reflect the structure of the music industry described in the second section. Specifically, in the case of *consumer spending*, we measure the subcategories: live music and recorded music. We further divide the recorded market into the subcategories: streaming, digital downloads, and physical (Table 3). First, the subcategory that reflects consumer spending on streaming shows an uptrend before and after the outbreak of COVID-19, increasing from a mean value of €2.34 per month in winter 2018/19 to a mean value of €3.14 per month in winter 2020/21. Next, the mean values for physical show a fairly constant downward trend independent of COVID-19, declining from €13.17 per month in winter 2018/19 to €8.06 per month in winter 2020/21. Surprisingly, the spending on digital downloads increases before the COVID-19 outbreak from €2.28 per month in winter 2018/19 to €3.27 per month in winter 2019/20, and then declines again to €2.12 in winter 2020/21 after the pandemic outbreak. This consumer spending data is in line with the IFPI global music report reflecting an increasing spending for streaming subscriptions, but declining spendings for digital and physical music [3]. The spending on live music again follows a seasonal pattern before the pandemic outbreak and then significantly declines from €14.84 per month in winter 2019/20 to €0.30 per month in winter 2020/21.

In the case of *music consumption*, we again include the subcategories: live music and recorded music. Similar to consumer spending, we further measure the subcategories: premium and free streaming, digital downloads, physical, radio, and online radio (Table 3). The mean values reveal that the total listening time across all formats decreases from 22.17 hours per week in winter 2018/19 to 19.00 hours per week in winter 2020/21, with the decline already starting before the COVID-19 outbreak in winter 2019/20 and continuing since then. This is in line with the IFPI report of 18.4 hours of consumer music listening per week [39]. While traditional radio is still by far the most popular format, it is also the main driver of the decline, dropping constantly before and after the pandemic outbreak from a mean of 10.45 hours per week in winter 2018/19 to a mean of 8.05 hours per week in winter 2020/21. On the contrary, the consumption of online radio is rather flat before the outbreak and then constantly increases from 2.94 hours per week in winter 2019/20 to 3.46 hours per week in winter 2020/21. Moreover, the mean values for premium streaming show a constant uptrend before and after the COVID-19 outbreak (1.67 hours per week in winter 2018/19 and 2.24 hours per week in winter 2020/21), while free streaming is rather constant before the outbreak and then starts to decline (0.81 hours per week in winter 2018/19 and 0.59 hours per week in winter 2020/21). Furthermore, the consumption of physical music and digital downloads slightly declines before the COVID-19 outbreak and is rather constant after the outbreak. Finally, a seasonal pattern in the consumption of live music events, with a higher consumption during the summer, is observable. The consumption of live music events dramatically drops after the pandemic outbreak from 0.32 hours per week in winter 2019/20 to 0.02 hours per week in winter 2020/21.

### Control variables

Table 4 shows the summary statistics for our control variables which can be divided into variables capturing music-related information from the respondents (i) as well as demographics.

**Table 4:** Control variables.

*Type*	*Variable*	*Description*	*Type*	*Pre-COVID outbreak*						*Post-COVID outbreak*							
				*Winter 2018/19*		*Summer 2019*		*Winter 2019/20*		*Summer 2020*		*Winter 2020/21*		*Total (all waves)*			
				*Mean*	*SD*	*Mean*	*SD*	*Mean*	*SD*	*Mean*	*SD*	*Mean*	*SD*	*Mean*	*SD*	*Min*	*Max*
Music-related control variables	MusicEducation	Musical education	Count	0.51	0.74	0.49	0.74	0.46	0.73	0.46	0.72	0.45	0.72	0.48	0.73	0.00	2.00
	MusicAppreciation	Appreciation of music	7-point Likert scale	4.31	1.80	4.24	1.84	4.26	1.84	4.19	1.91	4.21	1.91	4.24	1.86	1.00	7.00
	ActiveListening	Listen to music actively	7-point Likert scale	2.51	1.52	2.57	1.52	2.56	1.57	2.54	1.56	2.64	1.58	2.56	1.55	1.00	7.00
	MainstreamMusic	Listen to mainstream music	7-point Likert scale	3.58	1.51	3.59	1.51	3.66	1.54	3.66	1.57	3.69	1.52	3.63	1.53	1.00	7.00
	**Purchase reason**																
	PurchaseReason_Atmosphere	Atmosphere as the reason for purchase	Binary	0.12	0.32	0.14	0.35	0.12	0.33	0.07	0.26	0.07	0.25	0.10	0.30	0.00	1.00
	PurchaseReason_Flexibility	Flexibility as the reason for purchase	Binary	0.08	0.28	0.08	0.27	0.08	0.27	0.06	0.24	0.08	0.28	0.08	0.27	0.00	1.00
	PurchaseReason_Habit	Habit as the reason for purchase	Binary	0.20	0.40	0.20	0.40	0.19	0.40	0.19	0.40	0.18	0.39	0.19	0.39	0.00	1.00
	PurchaseReason_SoundQuality	Sound quality as the reason for purchase	Binary	0.14	0.35	0.13	0.33	0.10	0.30	0.13	0.33	0.13	0.33	0.12	0.33	0.00	1.00
	PurchaseReason_Mobility	Mobility as the reason for purchase	Binary	0.02	0.15	0.03	0.16	0.02	0.14	0.03	0.17	0.01	0.12	0.02	0.15	0.00	1.00
	PurchaseReason_None	No specific reason for purchase	Binary	0.20	0.40	0.17	0.37	0.19	0.39	0.18	0.39	0.19	0.39	0.19	0.39	0.00	1.00
	**Marital status**																
Demographics	MaritalStat_LivAlone	Living alone	Binary	0.33	0.47	0.34	0.47	0.34	0.47	0.34	0.48	0.34	0.47	0.34	0.47	0.00	1.00
	MaritalStat_LivAlone_Partner	Living alone and having a partner	Binary	0.08	0.27	0.06	0.24	0.07	0.26	0.07	0.26	0.08	0.27	0.07	0.26	0.00	1.00
	MaritalStat_LivTogether_Partner	Living together with your partner	Binary	0.60	0.49	0.60	0.49	0.59	0.49	0.58	0.49	0.58	0.49	0.59	0.49	0.00	1.00
	**Education**																
	**Education_NoSchool**	No school degree	Binary	0.01	0.08	0.00	0.06	0.01	0.07	0.01	0.07	0.00	0.06	0.00	0.07	0.00	1.00
	Education_ElementarySchool	Elementary school degree	Binary	0.10	0.30	0.10	0.30	0.10	0.30	0.10	0.30	0.10	0.30	0.10	0.30	0.00	1.00
	Education_MiddleSchool	Middle school degree	Binary	0.34	0.48	0.34	0.48	0.34	0.47	0.33	0.47	0.34	0.47	0.34	0.47	0.00	1.00
	Education_HighSchool	High school degree	Binary	0.20	0.40	0.19	0.39	0.19	0.39	0.19	0.40	0.20	0.40	0.19	0.40	0.00	1.00
	Education_Technical	Technical degree	Binary	0.07	0.25	0.07	0.25	0.08	0.27	0.07	0.26	0.05	0.23	0.07	0.25	0.00	1.00
	Education_Bachelors	Bachelors degree	Binary	0.07	0.25	0.07	0.25	0.07	0.26	0.08	0.27	0.07	0.26	0.07	0.26	0.00	1.00
	Education_Masters	Masters degree	Binary	0.21	0.41	0.21	0.41	0.21	0.41	0.21	0.40	0.22	0.42	0.21	0.41	0.00	1.00
	Education_PhD	PhD degree	Binary	0.01	0.09	0.01	0.09	0.01	0.09	0.01	0.09	0.01	0.09	0.01	0.09	0.00	1.00
	**Occupation**																
	Occupation_Unemployed	No profession	Binary	0.07	0.26	0.08	0.27	0.08	0.28	0.09	0.28	0.10	0.30	0.09	0.28	0.00	1.00
	Occupation_Homemaker	In charge of the household	Binary	0.08	0.27	0.08	0.27	0.07	0.25	0.06	0.24	0.06	0.25	0.07	0.26	0.00	1.00
	Occupation_School	Profession as a pupil	Binary	0.01	0.07	0.00	0.04	0.00	0.04	0.00	0.04	0.00	0.04	0.00	0.05	0.00	1.00
	Occupation_Apprenticeship	Profession in an apprenticeship	Binary	0.01	0.09	0.01	0.08	0.01	0.07	0.01	0.07	0.00	0.04	0.01	0.07	0.00	1.00
	Occupation_University	Profession as a student	Binary	0.03	0.17	0.02	0.15	0.02	0.15	0.02	0.14	0.02	0.12	0.02	0.15	0.00	1.00
	Occupation_Employed	Profession as an employee	Binary	0.64	0.48	0.63	0.48	0.64	0.48	0.63	0.48	0.62	0.49	0.63	0.48	0.00	1.00
	Occupation_Selfemployed	Profession as self-employed	Binary	0.06	0.24	0.07	0.26	0.07	0.25	0.07	0.26	0.07	0.26	0.07	0.25	0.00	1.00
	Occupation_Other	Other profession	Binary	0.10	0.30	0.11	0.31	0.11	0.32	0.12	0.32	0.12	0.33	0.11	0.32	0.00	1.00
	Gender_Female	Being female	Binary	0.42	0.49	0.42	0.49	0.42	0.49	0.42	0.49	0.42	0.49	0.42	0.49	0.00	1.00
	Age	Age in years	Continuous	48.60	11.68	49.13	11.62	49.54	11.61	50.02	11.63	50.45	11.61	49.55	11.64	17.00	70.00
	Children	Having children	Binary	0.54	0.50	0.54	0.50	0.54	0.50	0.54	0.50	0.55	0.50	0.54	0.50	0.00	1.00
	Income	Personal net income	Continuous	2121.62	1264.67	2237.33	1293.06	2227.20	1301.07	2243.24	1331.31	2288.85	1289.42	2223.65	1296.39	250.00	5250.00

Note: N = 2970 observations (594 respondents). Exact bi-annual data collection dates for our five periods are as follows: winter 2018/19 in January/February 2019, summer 2019 in July 2019, winter 2019/20 in January 2020, summer 2020 in June 2020, and winter 2020/21 in January 2021.

First, we measured the musical background and the consumption behavior of the respondents. The musical education variable reflects whether a participant plays an instrument and/or has ever had private music lessons. It is the sum of the two dummy indicators and can therefore reach a maximum of two. We include this variable as a proxy for the consumption capital of our respondents as those with a musical background have a higher stock of musical capital and thus a higher marginal utility for future music consumption [40]. While this variable declines before the pandemic outbreak from 0.51 in winter 2018/19 to 0.46 in winter 2020/21, it is rather constant after the outbreak. Next, we also include the appreciation of music to control for systematic differences within general music interest (e.g., [25]). This variable is measured on a seven-point Likert scale and shows a small downward trend before and after the COVID-19 outbreak declining from 4.31 in winter 2018/19 to 4.21 in winter 2020/21. Furthermore, we include a variable capturing if respondents actively listen to music as this indicates the level of involvement and the importance of music for the individual respondent, which might influence both consumption and spending behavior. We observe a slight uptrend for this variable continuing throughout the outbreak increasing from 2.51 in winter 2018/19 to 2.64 in winter 2020/21. In addition, we also include if a respondent listens to mainstream music as the level of supply might be different for mainstream and non-mainstream music, and consumers listening to mainstream genres might have a different listening and spending behavior (e.g., streaming vs. physical). We observe a slight uptrend for this variable increasing from 3.58 in winter 2018/19 to 3.69 in winter 2020/21. Finally, respondents were asked why they buy music via a specific channel as some specific channels are directly affected by restrictions after the outbreak–possible answers include: atmosphere, flexibility, habit, sound quality, mobility, or none/other. While most of the reasons are rather constant, the mean value for atmosphere drops after the outbreak (from 0.12 in winter 2018/19 to 0.07 in winter 2020/21).

Second, we control for heterogeneity and include relevant demographic data such as age, gender, children, income level, education, occupation, and marital status of respondents. Our average respondent is 49.55 years old and 54% of our respondents have children.

## Empirical results

**First**, we provide *model-free insights* on the development of spending and consumption behavior by analysing mean values within the observation period (Table 3). While consumers on average spent between €31.45 to €35.70 before the pandemic, consumers reduced their music spending down to €13.63 during the pandemic, resulting in a reduction of over 50%. Live spending particularly suffers from substantial losses during COVID-19. Whereas consumers’ live spending averaged between €14.20 to €14.54 during the pre-pandemic period, it drastically decreases to only €0.30 after the outbreak in winter 2020/21. Expenditures for the recorded market should be investigated more thoroughly. While consumers on average spent between €2.21 to €2.73 per month on streaming within the recorded market in the pre-pandemic period, spending increases since the pandemic started (winter 2020/21). In contrast, spending on physical and digital falls after the outbreak.

Surveyed data for the total music consumption reveals a continuous decline from 22.17 hours in winter 2018/19 to 19.00 hours in winter 2020/21. Due to the pandemic, live music consumption reduces to a minimum of 0.02 hours per week (winter 2020/21). The consumption via premium streaming demonstrates as being the biggest beneficiary in the recorded market with an increase from 1.67 hours in the initial observation period (winter 2018/19) to up to 2.24 hours two years later. Traditional radio, as the largest submarket for music consumption, continuously declines with 10.45 hours per week in the initial observation period down to 8.05 hours during the pandemic in winter 2020/21.

Second, we examine *model-based empirical results* by considering the COVID-19 effect on consumer spending and music consumption. As consumer spending and music consumption follow seasonal patterns (e.g., higher live music consumption during summer), we control for seasonality. In line with the previous structure, we first discuss the results of the log-linear fixed effects model for the total market (Table 5).

**Table 5:** Total market model.

*Variable*	*Consumer spending (ln)*				*Music consumption (ln)*			
	*Coefficient*		*SE*	*p-Value*	*Coefficient*		*SE*	*p-Value*
** COVID-19**	-0.495	***	0.053	0.000	-0.143	***	0.027	0.000
** Summer**	0.082	*	0.045	0.069	0.004		0.021	0.859
***Music-related control variables***								
MusicEducation	0.258	***	0.094	0.007	0.045		0.046	0.330
MusicAppreciation	0.013		0.028	0.638	0.023		0.014	0.105
ActiveListening	0.009		0.024	0.703	-0.021		0.013	0.116
MainstreamMusic	0.068	**	0.028	0.014	-0.018		0.016	0.273
** Purchase reason**								
PurchaseReason_Atmosphere	1.556	***	0.147	0.000				
PurchaseReason_Flexibility	0.823	***	0.131	0.000				
PurchaseReason_Habit	0.546	***	0.099	0.000				
PurchaseReason_SoundQuality	0.893	***	0.129	0.000				
PurchaseReason_Mobility	0.790	***	0.205	0.000				
PurchaseReason_Other	0.111		0.093	0.233				
***Demographics***								
** Marital status**								
MaritalStat_LivTogether_Partner	References				References			
MaritalStat_LivAlone	-0.137		0.247	0.580	0.144		0.128	0.264
MaritalStat_LivAlone_Partner	-0.029		0.236	0.902	-0.061		0.105	0.564
** Education**								
Education_MiddleSchool /Technical/HighSchool	References				References			
Education_NoSchool/ElementarySchool	0.109		0.273	0.689	-0.020		0.183	0.915
Education_Bachelors/Masters/PhD	-0.044		0.273	0.871	-0.023		0.072	0.745
** Occupation**								
Occupation_Employed/Selfemployed	References				References			
Occupation_Unemployed	0.103		0.202	0.612	-0.018		0.108	0.868
Occupation_Homemaker	0.201		0.229	0.379	-0.075		0.113	0.509
Occupation_School/Apprenticeship/University	0.343		0.335	0.307	-0.109		0.185	0.556
Occupation_Other	0.198		0.212	0.351	0.008		0.105	0.939
Age	-0.040	**	0.018	0.028	-0.014		0.011	0.189
Children	0.200		0.263	0.447	-0.021		0.130	0.875
Income (ln)	0.264	***	0.082	0.001	0.015		0.040	0.714
Overall R^2^	0.684				0.752			
Within R^2^	0.173				0.031			
Observations	2970				2960			

1) Note

* p < 0.1

**p < 0.05

***p < 0.01; N = 2970 observations (594 respondents) for consumer spending N = 2960 observations (592 respondents) for music consumption. For the analysis, we used balanced panel fixed effects estimation with robust standard errors in Stata 16.

Our empirical results reveal significant negative effects of the COVID-19 outbreak on music *consumer spending*. The results for our covariates are as expected, showing a positive and statistically significant (p < 0.01) association of musical education and personal net income with consumer spending. For *music consumption*, we observe a statistically significant downtrend in the post-pandemic period.

**Fig 1:**
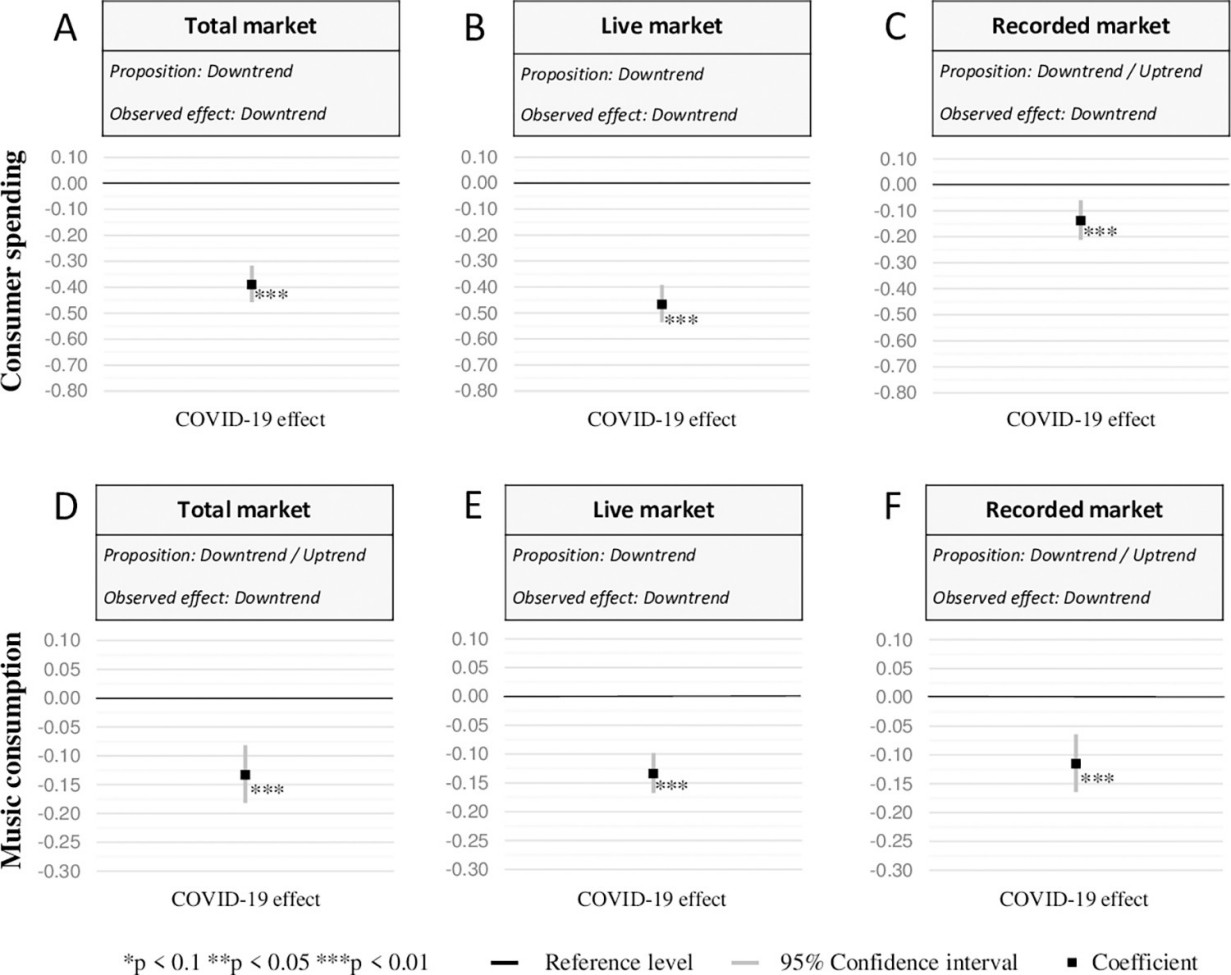
Effect sizes for the total market, the live market, and the recorded market. (A) The effect size of post-COVID outbreak period compared to the pre-COVID-19 period (reference level) for total consumer spending at a 95% confidence interval. Statistically significant changes are marked. The approach of Halvorsen and Palmquist [41] is used for the transformation of the coefficients of dummy variables. For the analysis, we used balanced panel fixed effects estimation with robust standard errors in Stata 16. (B) Same as (A) only for live consumer spending. (C) Same as (A) only for recorded consumer spending. (D) Same as (A) only for total music consumption. (E) Same as (A) only for live music consumption. (F) Same as (A) only for recorded music consumption.

**Fig 2:**
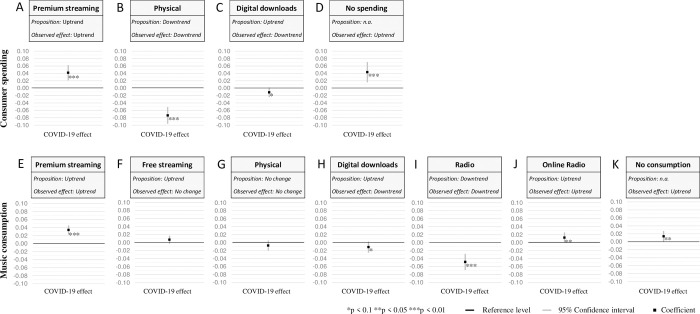
Average partial effect sizes for the submarkets of the recorded market. (A) The average partial effect size of the post-COVID-19 outbreak compared to pre-COVID-19 period for consumer spending on premium streaming at a 95% confidence interval. Statistically significant changes are marked. The fractional multinomial logit model with cluster-robust standard errors with respect to participants is estimated with STATA 16 and the module FMLOGIT [42]. (B) Same as (A) only for consumer spending on physical products. (C) Same as (A) only for consumer spending on digital downloads. (D) Same as (A) only for lack of consumer spending. (E) Same as (A) only for music consumption via premium streaming. (F) Same as (A) only for music consumption via free streaming. (G) Same as (A) only for music consumption via physical products. (H) Same as (A) only for music consumption via digital downloads. (I) Same as (A) only for music consumption via radio. (J) Same as (A) only for music consumption via online radio. (K) Same as (A) only for lack of music consumption.

Detailed empirical results for the live market as well as recorded market and its submarkets are provided in the Tables S1–S3 in S1 File. We derived Figs 1 and 2 as visual representations of our five panel variables in order to test our propositions.

We present the effect sizes across the total market, the live market, and the recorded market in Fig 1. We evaluate the impact of COVID-19 by using the dummy variable correction by Halvorsen and Palmquist [41] in a semilogarithmic regression equation with normal disturbances. This allows for the interpretation of the percentage change on (a) total consumer spending (in €) and (b) total music consumption (in hours). More precisely, the transformed coefficient of the respective dummy variable describes the percentage change in y associated with switching the dummy variable from 0 to 1, which is 100(*e*^*β*^−1).

Due to the COVID-19 outbreak, total market consumer spending decreases by 45.1% compared to the pre-pandemic period. The total music market consumption noticeably decreases by 13.3% after the COVID-19 outbreak.

Empirical results for consumer live music spending indicate strong declining expenditures by 46.8% caused by the pandemic. For live music consumption, we notice a significant reduction after the pandemic outbreak (13.4%).

Compared to the live music market, consumer spending on recorded music is less affected (-13.8%). The consumption of recorded music decreases during the outbreak by 11.5% even though consumers spent more time at home.

In summary, our empirical results indicate negative effects for the total market, the live market, and the recorded market from consumer spending and music consumption. Consequently, our propositions for the live market are confirmed by the empirical results formulated in Table 1, while we find clear negative effects for the total and recorded market.

Next, we present our results for the recorded submarkets in Fig 2. By estimating a fractional multinominal logit model, we reveal the changes of average partial effects compared to the pre-COVID period. Thus, the sub-analysis does not investigate the absolute change in total music spending (in €) and consumption (in hours) but does investigate the average percentage change in shares between the different channels from the recorded market. The overall Wald-χ^2^ test statistic for consumer spending is 732.30 (p<0.001) and 765.37 (p<0.001) for music consumption, both indicating a good explanatory power of the models (Table S3 in S1 File).

The share of consumer spending on premium streaming substantially increases with the outbreak of the pandemic (4.2%). In contrast, the share of consumer spending on physical music strongly decreases after the outbreak (-7.4%). These results are in line with our initial propositions. Consumer spending on digital downloads does not benefit from the pandemic and slightly decreases after the COVID-19 outbreak (-1.1%). Furthermore, we also control for participants without any music spending. The share of consumers without any spending substantially increased (4.3%).

With the outbreak of COVID-19, premium streaming consumption increases by 3.4% while we observe no significant change for free streaming or physical music consumption. In contrast to traditional radio (-4.9%), the share of music consumption via online radio significantly increases due to the pandemic outbreak (1.2%). Overall, these observations confirm our propositions. However, free streaming and digital downloads from digital channels do not benefit from the pandemic as expected, with digital downloads even showing significant negative effects after the pandemic outbreak (-1.1%). Finally, we find positive significant effects for participants with no consumption during the observed period.

To check the robustness of our findings, we also estimated alternative variants of all our models: (a) weighted balanced models (Tables S6-S9 in S1 File), (b) models using unbalanced data (Tables S6-S9 in S1 File), and (c) for the market-level models fixed versus random effects models (Tables S10-S12 in S1 File). Overall, our post-COVID-19 variables’ results are highly robust. All variants from our main model confirm the sign of the coefficients and the statistical significance of our findings.

We further analyzed different subsamples separately to capture specific individual heterogeneity. To test differences in coefficients between subsamples, we next include interactions of the grouping dummy variable, with all our independent variables in our full models. While the main effect of the grouping dummy is omitted in the fixed effects models, differences between our subsamples are indicated by the interactions. The results for the investigated sub-samples and differences analysis between the subsamples are presented in (Table S13 in S1 File).

First, we analyzed subsamples based on socio-demographics capturing differences between gender and age based on median split. However, differences between the subsamples are not significant in the post-COVID-19 effect, indicating no differences between male and female, nor for younger and older participants, due to the COVID-19 pandemic.

Next, we investigate music-related subsamples such as high versus low music appreciation and high versus low music education. We expect individuals with a high music appreciation and education to experience a higher involvement in music, which could lead to more music consumption and spending than individuals with low music appreciation and education. Thus, we expect individuals with high music appreciation and education to be conceivably affected in terms of consumer spending and music consumption due to the pandemic outbreak.

For music appreciation, we observe significant differences between high and low appreciation for consumer spending in all panel models. For the subsample of high music appreciation, the outbreak of COVID-19 has a stronger negative effect on consumer spending than the comparative group with low music appreciation.

For music education, we observe significant differences in consumer spending and music consumption in the live market. The subsample with higher music education reduces its live spending and music consumption stronger than the comparative group with a less musical education background.

## Discussion

### Implications

This research provides new and important insights into the effect of an exogenous shock on the music industry. Ex ante, the total effects on spending–particularly listening behavior–are rather unclear as specific channels benefit from the pandemic outbreak (e.g., digital channels), while other channels clearly suffer (e.g., live music). Our panel study design allows us to quantify the COVID-19 effects and to analyze general industry trends while controlling for covariates.

#### Total market

As indicated by our results, total *consumer spending* declines after the pandemic outbreak. The pandemic contributes to a decline in physical spending (e.g., due to closed music stores and frictions in the supply chain) and live spending (e.g., due to the ban of physical live events). Overall, the results reveal a decreas**e** in consumer spending of more than 45% compared to pre-pandemic.

For *music consumption*, the results show a decrease after the outbreak of COVID-19. Explanatory statements for the decline might be attributable to competing entertainment opportunities such as video streaming and social media platforms. Hence, the music industry needs to adapt its offer to the digital natives’ generations. Especially because of the development during the lockdown when consumers spent a lot of time at home and revealed the concerning insight that music consumption depends on mobility and social occasions.

#### Live market

Since physical live events are subject to governmental restrictions during the pandemic, *consumer spending* and *consumption* has respectively and significantly decreased. Decreasing merchandise revenues due to a lack, or cancellation, of tours might additionally lead to a decline in consumer spending. Even in winter 2020/21, consumer spending on live music was close to zero (Table 3) reflecting both health and safety concerns as well as regulatory restrictions.

If the restrictions on live music events continue, artists might further strengthen their live music streams on digital platforms such as YouTube and Twitch. According to our ongoing online panel survey, 34% of respondents have already watched a live music stream with an average willingness to pay of €7.10 in winter 2020/21. However, this new income stream has not been sufficient in preventing the collapse of live music. In the long-term, broadcasting formats have the potential to complement physical live events as the pandemic might have sustainably shaped consumer behavior by establishing and increasing the willingness to pay for digital content. Emerging online live events could also open up the opportunity for hybrid live event formats which would increase audience potential.

Moreover, the pandemic outbreak has once again shown artists’ dependence on live music events as a major source of income. Accordingly, labels and artists should be striving to improve potential monetisation from online (live) platforms [43]. Furthermore, artists might want to consider negotiating contracts that allow labels to participate in live music revenues in exchange for a more favorable distribution of the stable, recorded music revenues.

In addition, ticketers and event organizers face a challenging situation. It is unclear when the live market will revitalize. Once the market recovers, it will be crucial to find enough locations to serve the demand while complying with potential COVID-19 restrictions, such as the obligation to test concert attendees for COVID-19 or to validate attendees’ vaccination status.

#### Recorded market

We analyze the sublevels of the recorded market and their differing developments below.

*Premium streaming*: Premium streaming spending has strongly increased as the current crisis contributes to the shift from physical to digital music. Thus, streaming platforms such as Spotify, Apple Music, and Amazon Music benefit from the COVID-19 pandemic because they implemented digital-based revenue models that are not exposed to frictions in the supply chain. Throughout the pandemic, subscription revenues have shown to be a robust revenue stream. Moreover, once customer lock-in effects are realized, streaming services could even increase their average revenue by increasing the monthly subscription fee as Netflix has in the video streaming industry [44].

*Free streaming*: While premium streaming has strongly benefited from the pandemic outbreak, the consumption of free music streaming remains at a moderate level during the observation period. Contrary to our propositions, this channel did not benefit from the digitalization of the music industry during the pandemic. As consumers increasingly decide to consume and spend on premium over free music streaming, streaming platforms might strategically focus their efforts on premium users that generate higher revenues for the music industry. However, free streaming helps consumers explore music beyond already well-known established artists representing a beneficial entry channel for up-and-coming artists and their corresponding labels [45].

*Physical*: Consumer spending on physical products decisively decreased in the beginning of the pandemic with the lockdown resulting in store closures or click and collect options that largely eliminated the opportunity to purchase physical music products. As spending on physical music products decreases, labels and artists should look into which type of consumer it is still viable to produce physical albums for as the pandemic has accelerated the digitalization of music because consumers value the convenience of streaming services.

*Digital downloads*: We investigate the decrease in spending and consumption on digital downloads. Streaming might be more attractive for consumers as it provides access to a large catalogue of music while needing less storage capacity (particularly relevant for mobile phone users). In addition, it offers consumer friendly subscription-based pricing models. Therefore, the channel for digital downloads might be increasingly substituted by streaming.

*Radio*: The consumption of traditional radio stations faces a major a challenge since the outbreak of COVID-19. Confinement measures have led to a significant decrease in commute listening and music consumption at the workplace, significantly reducing radio music consumption post-outbreak. Additionally, decreasing music consumption in shops, bars, and gyms has also negatively affected radio music consumption.

*Online Radio*: The increase in time spent at home may explain the rising online radio consumption as it may potentially substitute the traditional radio station. However, as users might access online radio via third party applications, radio stations might lose the direct interface to its consumers which could in turn lead to losing advertising control.

In conclusion, the submarket analysis reveals the pandemic as an accelerator of industry trends and changing consumer behavior. Despite the decreasing consumer spending on recorded music during the pandemic, the music industry has mastered a transition from physical sales to online subscription-based services. Regarding the live market, new revenue streams such as live online events have emerged and might be established as complementary revenue streams.

## Limitations and future research

Our study has several limitations. First, surveyed consumers’ preferences depend, in terms of validity, on the optimal recall length in survey design and consumers’ ability to accurately recall behavior [46]. Consequently, we validated our surveyed data on consumer spending and music consumption with well-established music market reports which support the reliability of our data. Second, we rely on stated behavior from five online surveys, which could be enriched if streaming services and labels share their user data. However, matching that data with data on live music events would still be challenging. Third, our sample only relies on respondents from the German music market. Fourth, our data deals with music consumption and spending on the music market and does not include non-market consumer behavior (e.g., private music events). Fifth, our balanced panel is not fully representative as especially younger and female respondents dropped out in later waves. However, estimation results remain robust when estimating a fixed effects panel model with robust standard errors weighted by a sampling weight to correct for the loss of respondents with specific combinations of age and gender (Tables S6-S9 in File S1). Sixth, our data allows no conclusion on whether effects are driven by the supply or demand side. For example, the decline in physical sales could be due to a lack of supply because of delayed albums or frictions in the supply chain, but could also be due to consumers shifting to a digital channel. However, we can show the trend before the pandemic outbreak which allows us to separate the general trend from the COVID-19 effect.

We hope that the results of our study will spark future research within the entertainment industry. Future studies might extend our analysis to include other countries and analyze the recovery of the music industry after the pandemic. Moreover, future research could also analyze if COVID-19 trends, such as accelerated digitalization, are sustained or reversed in the long-term after restrictions on public life are lifted.

## Supporting information

S1 File (PDF)
