# Supplementary material for: The impact of COVID-19 on music consumption and music spending
Source: PLoS One. 2022 May 13;17(5):e0267640. doi: 10.1371/journal.pone.0267640 (PMC9106175; doi:10.1371/journal.pone.0267640)
Supplement: S1 File — (PDF) [file pone.0267640.s001.pdf]

Supporting Information for

The Impact of COVID-19 on Music Consumption and Music Spending

Janis Denk, Alexa Burmester, Michael Kandziora, Michel Clement

Correspondence to: michel.clement@uni-hamburg.de

This file includes:

Supplementary information text

Live, recorded, and recorded submarket models (Tables S1 – S3)

Online study on music usage (relevant excerpt) (Table S4)

Sample representativity (Table S5)

Fixed effects, fixed effects weighted, and fixed effects unbalanced model (Tables S6 – S9)

Random effects model (Tables S10 – S12)

Subsample models (Table S13)

Supplementary Information Text

Method. To analyze consumers' pre- and post-pandemic shifts in consumer spending (in euros) and music consumption (in hours), we used the STATA 16 program. Furthermore, we used the STATA module FMLOGIT for the estimation of the fractional multinomial logit model [42].

Table S1. Live Market Model

Variable	*Consumer spending (ln)*				*Music consumption (ln)*			
	Coefficient		*SE*	*p-Value*	*Coefficient*		*SE*	*p-Value*
COVID-19	-0.630	***	0.061	0.000	-0.143	***	0.018	0.000
Summer	0.146	***	0.040	0.000	0.058	***	0.013	0.000
Music related control variables								
MusicEducation	0.263	***	0.092	0.004	-0.018		0.032	0.584
MusicAppreciation	-0.031		0.026	0.234	0.006		0.007	0.379
ActiveListening	0.009		0.021	0.665	0.004		0.007	0.624
MainstreamMusic	0.048	*	0.029	0.095	-0.007		0.008	0.364
**Purchase reason**								
PurchaseReason_Atmosphere	1.489	***	0.152	0.000				
PurchaseReason_Flexibility	-0.025		0.113	0.827				
PurchaseReason_Habit	0.220	**	0.088	0.013				
PurchaseReason_SoundQuality	0.337	***	0.113	0.003				
PurchaseReason_Mobility	-0.067		0.193	0.728				
PurchaseReason_Other	0.002		0.079	0.983				
Demographics								
**Marital status**								
MaritalStat_LivTogether_Partner	References				References			
MaritalStat_LivAlone	0.048		0.267	0.856	-0.005		0.082	0.952
MaritalStat_LivAlone_Partner	0.114		0.235	0.628	-0.012		0.066	0.851
**Education**								
Education_MiddleSchool/Technical/HighSchool	References				References			
Education_NoSchool/ElementarySchool	-0.008		0.251	0.973	0.024		0.118	0.837
Education_Bachelors/Masters/PhD	-0.073		0.229	0.751	-0.002		0.067	0.979
**Occupation**								
Occupation_Employed/Selfemployed	References				References			
Occupation_Unemployed	0.152		0.161	0.345	0.031		0.040	0.431
Occupation_Homemaker	0.220		0.184	0.232	0.067		0.051	0.189
Occupation_School/Apprenticeship/University	0.427		0.269	0.113	0.095		0.126	0.453
Occupation_Other	0.208		0.160	0.196	0.016		0.049	0.751
Age	-0.009		0.029	0.763	-0.002		0.008	0.842
Children	0.342	*	0.192	0.075	-0.012		0.078	0.875
Income (ln)	0.121	*	0.070	0.082	-0.015		0.024	0.545
Overall R^2	0.503				0.429			
Within R^2	0.206				0.056			
Observations	2,970				2,960			

Note: * p < 0.1; **p < 0.05; ***p < 0.01; N = 2970 observations (594 respondents) for consumer spending N = 2960 observations (592 respondents) for music consumption. For the analysis, we used balanced panel fixed effects estimation with robust standard errors in Stata 16.

Table S2. Recorded Market Model

Variable	*Consumer spending (ln)*				*Music consumption (ln)*			
	Coefficient		*SE*	*p-Value*	*Coefficient*		*SE*	*p-Value*
COVID-19	-0.149	***	0.041	0.000	-0.123	***	0.027	0.000
Summer	-0.037		0.036	0.308	-0.005		0.021	0.821
Music related control variables								
MusicEducation	0.120		0.074	0.104	0.048		0.045	0.287
MusicAppreciation	0.053	**	0.023	0.018	0.024		0.015	0.106
ActiveListening	-0.003		0.022	0.875	-0.023	*	0.013	0.074
MainstreamMusic	0.021		0.023	0.363	-0.019		0.016	0.235
**Purchase reason**								
PurchaseReason_Atmosphere	0.306	***	0.103	0.003				
PurchaseReason_Flexibility	0.712	***	0.110	0.000				
PurchaseReason_Habit	0.374	***	0.079	0.000				
PurchaseReason_SoundQuality	0.670	***	0.110	0.000				
PurchaseReason_Mobility	0.841	***	0.192	0.000				
PurchaseReason_Other	0.050		0.069	0.470				
Demographics								
**Marital status**								
MaritalStat_LivTogether_Partner	References				References			
MaritalStat_LivAlone	-0.136		0.215	0.529	0.153		0.129	0.234
MaritalStat_LivAlone_Partner	-0.154		0.204	0.449	-0.059		0.107	0.585
**Education**								
Education_MiddleSchool/Technical/HighSchool	References				References			
Education_NoSchool/ElementarySchool	0.004		0.190	0.983	-0.025		0.175	0.886
Education_Bachelors/Masters/PhD	-0.048		0.214	0.823	-0.033		0.070	0.636
**Occupation**								
Occupation_Employed/Selfemployed	References				References			
Occupation_Unemployed	-0.019		0.163	0.907	-0.016		0.109	0.884
Occupation_Homemaker	-0.001		0.175	0.994	-0.088		0.113	0.440
Occupation_School/Apprenticeship/University	0.074		0.218	0.735	-0.150		0.170	0.380
Occupation_Other	0.022		0.155	0.885	0.004		0.106	0.971
Age	-0.037	***	0.014	0.006	-0.013		0.010	0.180
Children	-0.033		0.227	0.883	-0.017		0.130	0.896
Income (ln)	0.198	***	0.072	0.006	0.022		0.039	0.582
Overall R^2	0.720				0.756			
Within R^2	0.069				0.027			
Observations	2960				2960			

Note: * p < 0.1; **p < 0.05; ***p < 0.01; N = 2970 observations (594 respondents) for consumer spending N = 2960 observations (592 respondents) for music consumption.
For the analysis, we used balanced panel fixed effects estimation with robust standard errors in Stata 16.

Table S3. Recorded Submarket Model

Variable	Consumer spending											
	Premium streaming			*Physical*			*Digital downloads*			*No spending*		
	APE	*SE*	*p-Value*	*APE*	*SE*	*p-Value*	*APE*	*SE*	*p-Value*	*APE*	*SE*	*p-Value*
COVID-19	0.042 ***	0.010	0.000	-0.074 ***	0.011	0.000	-0.011 *	0.006	0.058	0.043 ***	0.013	0.001
Summer	-0.006	0.008	0.481	0.004	0.011	0.703	-0.002	0.006	0.758	0.003	0.012	0.768
Music related control variables												
MusicEducation	0.018	0.014	0.196	0.012	0.011	0.265	0.002	0.007	0.818	-0.032 *	0.016	0.052
MusicAppreciation	0.011 **	0.005	0.034	0.047 ***	0.006	0.000	0.011 ***	0.004	0.005	-0.069 ***	0.006	0.000
ActiveListening	-0.016 ***	0.005	0.003	0.002	0.005	0.732	0.002	0.004	0.519	0.012 *	0.007	0.088
MainstreamMusic	0.019 ***	0.006	0.003	-0.002	0.006	0.773	-0.001	0.004	0.695	-0.016 **	0.008	0.038
**Purchase reason**												
PurchaseReason_Atmosphere	-0.165 ***	0.034	0.000	0.317 ***	0.058	0.000	0.214 ***	0.056	0.000	-0.366 ***	0.054	0.000
PurchaseReason_Flexibility	0.003	0.028	0.915	0.296 ***	0.059	0.000	0.336 ***	0.058	0.000	-0.635 ***	0.050	0.000
PurchaseReason_Habit	-0.151 ***	0.028	0.000	0.292 ***	0.057	0.000	0.278 ***	0.057	0.000	-0.418 ***	0.049	0.000
PurchaseReason_SoundQuality	-0.145 ***	0.030	0.000	0.341 ***	0.058	0.000	0.285 ***	0.057	0.000	-0.481 ***	0.052	0.000
PurchaseReason_Mobility	0.027	0.039	0.490	0.286 ***	0.067	0.000	0.315 ***	0.061	0.000	-0.629 ***	0.069	0.000
PurchaseReason_Other	-0.180 ***	0.030	0.000	0.213 ***	0.058	0.000	0.259 ***	0.058	0.000	-0.292 ***	0.051	0.000
Demographics												
**Marital status**												
MaritalStat_LivTogether_Partner	Reference			Reference			Reference			Reference		
MaritalStat_LivAlone	-0.011	0.024	0.664	-0.009	0.021	0.671	0.020	0.014	0.152	-0.001	0.029	0.984
MaritalStat_LivAlone_Partner	-0.021	0.037	0.569	0.035	0.038	0.354	-0.013	0.022	0.548	-0.002	0.043	0.964
**Education**												
Education_MiddleSchool/Technical/HighSchool	Reference			Reference			Reference			Reference		
Education_NoSchool/ElementarySchool	0.021	0.039	0.592	-0.013	0.033	0.699	0.015	0.029	0.594	-0.023	0.044	0.601
Education_Bachelors/Masters/PhD	-0.005	0.023	0.836	0.017	0.021	0.410	-0.011	0.012	0.352	-0.001	0.028	0.971
**Occupation**												
Occupation_Employed/Selfemployed	Reference			Reference			Reference			Reference		
Occupation_Unemployed	-0.056	0.034	0.105	0.020	0.042	0.627	-0.034 **	0.016	0.034	0.070	0.045	0.120
Occupation_Homemaker	-0.023	0.033	0.492	-0.009	0.033	0.775	-0.031 *	0.017	0.070	0.062	0.042	0.138
Occupation_School/Apprenticeship/University	0.051	0.059	0.389	-0.053	0.054	0.333	-0.005	0.030	0.856	0.006	0.072	0.931
Occupation_Other	0.011	0.038	0.771	-0.012	0.029	0.670	-0.012	0.025	0.640	0.014	0.044	0.754
GenderFemale	-0.057 ***	0.022	0.010	-0.031	0.019	0.109	0.003	0.013	0.804	0.084 ***	0.026	0.001
Age	-0.004 ***	0.001	0.000	0.002 *	0.001	0.058	-0.001	0.001	0.128	0.003 ***	0.001	0.007
Children	-0.002	0.023	0.946	0.026	0.020	0.200	-0.006	0.012	0.628	-0.019	0.027	0.493
Income (ln)	0.025	0.017	0.148	-0.022	0.015	0.131	0.016 *	0.009	0.065	-0.019	0.020	0.346
Wald chi^2	732.30											
Prob > chi^2	0.000											
Observations	2,970											

Note: * $p < 0.1$; ** $p < 0.05$; *** $p < 0.01$; N = 2970 observations (594 respondents) for consumer spending. For the analysis, we used a fractional multinomial logit model and report average partial effects with clustered standard errors by participant. The fractional multinomial logit model is estimated with the STATA 16 and the module FMLOGIT [42].

Table S3. (cont'd) Recorded Submarket Model

Variable	Music consumption											
	Premium streaming				*Free streaming*				*Physical*			
	APE		*SE*	*p-Value*	*APE*		*SE*	*p-Value*	*APE*		*SE*	*p-Value*
COVID-19	0.034	***	0.005	0.000	0.007		0.004	0.101	-0.007		0.006	0.187
Summer	-0.005		0.004	0.237	-0.002		0.003	0.549	0.006		0.005	0.196
Music related control variables												
MusicEducation	0.006		0.008	0.416	0.004		0.006	0.488	0.005		0.009	0.568
MusicAppreciation	0.006	*	0.003	0.054	-0.007	***	0.002	0.000	0.031	***	0.004	0.000
ActiveListening	-0.010	***	0.004	0.007	0.001		0.002	0.736	0.010	***	0.004	0.007
MainstreamMusic	0.005		0.004	0.241	-0.001		0.002	0.548	-0.019	***	0.004	0.000
Demographics												
**Marital status**												
MaritalStat_LivTogether_Partner	Reference				Reference				Reference			
MaritalStat_LivAlone	-0.001		0.016	0.956	0.018	*	0.011	0.085	0.010		0.016	0.533
MaritalStat_LivAlone_Partner	0.004		0.028	0.878	-0.002		0.013	0.879	0.011		0.024	0.639
**Education**												
Education_MiddleSchool/Technical/HighSchool	Reference				Reference				Reference			
Education_NoSchool/ElementarySchool	0.052	*	0.031	0.090	0.006		0.014	0.649	0.010		0.023	0.668
Education_Bachelors/Masters/PhD	-0.008		0.014	0.578	0.016		0.011	0.136	0.026		0.016	0.113
**Occupation**												
Occupation_Employed/Selfemployed	Reference				Reference				Reference			
Occupation_Unemployed	-0.052	***	0.014	0.000	0.009		0.016	0.586	0.017		0.020	0.391
Occupation_Homemaker	0.019		0.026	0.479	-0.012		0.013	0.377	0.011		0.026	0.675
Occupation_School/Apprenticeship/University	0.099	*	0.057	0.081	0.032		0.025	0.194	0.026		0.045	0.564
Occupation_Other	-0.001		0.029	0.973	0.013		0.015	0.388	-0.009		0.018	0.604
GenderFemale	-0.032	**	0.015	0.038	-0.010		0.009	0.275	0.030	**	0.014	0.031
Age	-0.004	***	0.001	0.000	-0.002	***	0.000	0.001	0.003	***	0.001	0.000
Children	0.009		0.014	0.536	-0.015		0.010	0.112	0.005		0.015	0.741
Income (ln)	0.015		0.010	0.127	0.002		0.004	0.690	-0.008		0.009	0.359
Wald chi^2	765.37											
Prob > chi^2	0.000											
Observations	2960											

Note: * p < 0.1; **p < 0.05; ***p < 0.01; N = 2960 observations (592 respondents) for music consumption. For the analysis, we used a fractional multinomial logit model and report average partial effects with cluster-robust standard errors with respect to participants. The fractional multinomial logit model is estimated with the STATA 16 and the module FMLOGIT [42].

Table S3. (cont'd) Recorded Submarket Model

Variable	*Music consumption*											
	Digital downloads			*Radio*			*Online Radio*			*No consumption*		
	APE	*SE*	*p-Value*	*APE*	*SE*	*p-Value*	*APE*	*SE*	*p-Value*	*APE*	*SE*	*p-Value*
COVID-19	-0.011 *	0.006	0.076	-0.049 ***	0.010	0.000	0.012 **	0.006	0.033	0.014 **	0.006	0.023
Summer	0.000	0.005	0.980	0.013	0.008	0.113	-0.008	0.005	0.127	-0.004	0.005	0.358
Music related control variables												
MusicEducation	0.005	0.009	0.622	-0.009	0.014	0.542	-0.014	0.010	0.151	0.003	0.006	0.696
MusicAppreciation	0.011 ***	0.004	0.003	-0.028 ***	0.005	0.000	-0.002	0.004	0.557	-0.011 ***	0.003	0.000
ActiveListening	0.009 **	0.004	0.034	-0.013 **	0.006	0.032	-0.006 *	0.004	0.100	0.009 ***	0.003	0.000
MainstreamMusic	-0.021 ***	0.005	0.000	0.033 ***	0.007	0.000	0.008 *	0.004	0.069	-0.004	0.003	0.149
Demographics												
**Marital status**												
MaritalStat_LivTogether_Partner	Reference			Reference			Reference			Reference		
MaritalStat_LivAlone	0.034 **	0.017	0.050	-0.076 ***	0.026	0.003	0.027 **	0.011	0.013	0.027 **	0.011	0.013
MaritalStat_LivAlone_Partner	0.028	0.030	0.364	-0.027	0.042	0.520	0.002	0.016	0.919	0.002	0.016	0.919
**Education**												
Education_MiddleSchool/Technical/HighSchool	Reference			Reference			Reference			Reference		
Education_NoSchool/ElementarySchool	-0.030	0.021	0.145	-0.067 *	0.038	0.078	0.019	0.015	0.203	0.019	0.015	0.205
Education_Bachelors/Masters/PhD	0.001	0.017	0.968	-0.040 *	0.024	0.097	-0.011	0.008	0.197	-0.011	0.008	0.197
**Occupation**												
Occupation_Employed/Selfemployed	Reference			Reference			Reference			Reference		
Occupation_Unemployed	0.073 **	0.036	0.041	-0.022	0.039	0.578	-0.030	0.022	0.176	0.004	0.013	0.746
Occupation_Homemaker	-0.003	0.031	0.915	0.008	0.046	0.862	-0.035	0.023	0.123	0.013	0.017	0.459
Occupation_School/Apprenticeship/University	-0.051 **	0.024	0.032	-0.058	0.068	0.387	-0.035	0.043	0.426	-0.013	0.014	0.347
Occupation_Other	0.006	0.023	0.788	-0.015	0.035	0.674	-0.021	0.021	0.313	0.026	0.018	0.148
GenderFemale	-0.029 **	0.014	0.038	0.087 ***	0.023	0.000	-0.045 ***	0.016	0.005	-0.001	0.009	0.952
Age	-0.004 ***	0.001	0.000	0.005 ***	0.001	0.000	0.001	0.001	0.384	-0.000	0.000	0.891
Children	-0.018	0.014	0.221	0.019	0.024	0.429	0.002	0.016	0.885	-0.002	0.010	0.832
Income (ln)	0.001	0.011	0.905	0.015	0.016	0.336	-0.019 *	0.011	0.100	-0.007	0.006	0.271
Wald chi^2	765.37											
Prob > chi^2	0.000											
Observations	2960											

Note: * $p < 0.1$; ** $p < 0.05$; *** $p < 0.01$; N = 2960 observations (592 respondents) for music consumption. For the analysis, we used a fractional multinomial logit model and report average partial effects with cluster-robust standard errors with respect to participants. The fractional multinomial logit model is estimated with the STATA 16 and the module FMLOGIT [42].

Table S4. Online study on music usage (relevant excerpt).

Variable	*Questionnaire*
Spending	How much money (euros) have you spent on music in the last 30 days? Please indicate your expenses in €.<br><br>Physical:<br>o CD albums<br>o CD singles<br>o Music DVDs / Blu-Rays<br>o Vinyl albums<br>o Deluxe boxes (music product +merchandising articles)<br><br>Digital downloads:<br>o Paid digital album downloads (whole albums only) (e.g., iTunes, Musicload, Amazon MP3)<br>o Paid digital track downloads (only individual music tracks) (e.g., iTunes, Musicload, Amazon MP3)<br><br>Streaming:<br>o Digital music subscription services (e.g., Spotify Premium, Apple Music, Musicload Nonstop, Amazon Music, Amazon Music Unlimited)<br>o Other digital music subscription services (e.g., Flatster, ZEEZEE, Musicmonster)<br><br>Live:<br>o Concerts (live music events)<br>o Festivals (live music events)<br>o Club concerts (live music events)
Consumption	How many hours have you listened to music over the last seven days in the following formats?<br>o Conventional radio (e.g., in the car, at work, at home)<br>o Online radio<br>o Physical music devices (e.g., CD / Vinyl / DVD)<br>o Digital downloaded music files on your devices (e.g., PC / Mac, MP3 player, smartphone, tablet)<br>o Free music streaming services (only the free ones with commercial breaks)<br>o Paid (subscription) music streaming services (e.g., Spotify Premium, Deezer Premium, Apple Music, Amazon Music, etc.)<br>o Live music
Gender	Please indicate your gender.<br>o Female<br>o Male<br>o Diverse
Age	Please indicate your age in years.

Marital status	Please indicate your relationship status.<br>o Living together with your partner<br>o Living alone<br>o Living alone and having a partner
Children	Do you have children?<br>o Yes<br>o No
Education	What is your highest education level?<br>o No school<br>o Elementary school diploma<br>o Middle school diploma<br>o High school diploma<br>o Technical degree<br>o Bachelor's degree<br>o Master's degree<br>o PhD degree
Occupation	Which of the following best describes your employment type?<br>o Pupil<br>o Apprentice<br>o Student<br>o Employee<br>o Self-employed<br>o Homemaker<br>o Unemployed<br>o Other
Income	Please state your personal monthly net income in euros.<br>o Less than 500 euros<br>o 501 to 1,000 euros<br>o 1,001 to 1,500 euros<br>o 1,501 to 2,000 euros<br>o 2,001 to 2,500 euros<br>o 2,501 to 3,000 euros<br>o 3,001 to 3,500 euros<br>o 3,501 to 4,000 euros<br>o 4,001 to 4,500 euros<br>o 4,501 to 5,000 euros<br>o More than 5,000 euros
Music education (1/2)	What instruments do you play?<br>o Guitar<br>o Piano/Keyboard<br>o Drums<br>o Violin<br>o Cello<br>o Bass<br>o Wind instrument<br>o Other<br>o None

Music education (2/2)	Have you ever regularly taken private music lessons for more than one year?<br>o Yes<br>o No
Active listening	When I listen to music, I don't do anything else. (7-point Likert scale)
Mainstream music	Would you classify your musical taste as more mainstream (i.e., current charts, musical taste of the masses) or non-mainstream? (7-point Likert scale)
Music appreciation	Music is always valuable to me so I like to spend money on music products. (7-point Likert scale)
Purchase reason	Why do you often buy the music you mentioned above the most?<br>o Habit<br>o Mobility<br>o Sound quality<br>o Atmosphere<br>o Flexibility<br>o No specific reason
Willingness to pay	How much are you willing to pay (in euros) for the following live music events by an artist you are interested in? Please indicate willingness to pay in euros.<br>o A car concert<br>o A live internet stream

Table S5. Sample representativity

	Target population Germany (in %)	*Full Sample (in %)*
Female gender	50	49
Age groups		
20-29	18	15
30-39	19	17
40-49	19	21
50-59	25	24
60-69	19	20

Note: Target population data in Germany refer to Bundeszentrale für politische Bildung [38].
The sum of the full sample does not add to 100% as we also have age groups younger than 20 and older than 69 years.

Table S6. Total Market Model (robustness check)

Variable	Consumer spending											
	Balanced fixed effects (model 1)				*Weighted fixed effects (model 2)*				*Unbalanced fixed effects (model 3)*			
	Coefficient		*SE*	*p-Value*	*Coefficient*		*SE*	*p-Value*	*Coefficient*		*SE*	*p-Value*
COVID-19	-0.495	***	0.053	0.000	-0.497	***	0.058	0.000	-0.435	***	0.033	0.000
Summer	0.082	*	0.045	0.069	0.104	**	0.053	0.049	0.024		0.026	0.352
Music related control variables												
MusicEducation	0.258	***	0.094	0.007	0.293	***	0.093	0.002	0.219	***	0.053	0.000
MusicAppreciation	0.013		0.028	0.638	0.018		0.029	0.535	0.052	***	0.016	0.001
ActiveListening	0.009		0.024	0.703	0.023		0.026	0.379	0.002		0.014	0.909
MainstreamMusic	0.068	**	0.028	0.014	0.073	**	0.032	0.022	0.028	*	0.016	0.081
**Purchase reason**												
PurchaseReason_Atmosphere	1.556	***	0.147	0.000	1.555	***	0.168	0.000	1.751	***	0.082	0.000
PurchaseReason_Flexibility	0.823	***	0.131	0.000	0.917	***	0.147	0.000	0.797	***	0.076	0.000
PurchaseReason_Habit	0.546	***	0.099	0.000	0.584	***	0.104	0.000	0.673	***	0.058	0.000
PurchaseReason_SoundQuality	0.893	***	0.129	0.000	0.967	***	0.146	0.000	1.092	***	0.076	0.000
PurchaseReason_Mobility	0.790	***	0.205	0.000	0.783	***	0.206	0.000	0.869	***	0.111	0.000
PurchaseReason_Other	0.111		0.093	0.233	0.150		0.094	0.112	0.208	***	0.055	0.000
Demographics												
**Marital status**												
MaritalStat_LivTogether_Partner		References				References				References		
MaritalStat_LivAlone	-0.137		0.247	0.580	-0.087		0.275	0.751	-0.116		0.122	0.341
MaritalStat_LivAlone_Partner	-0.029		0.236	0.902	0.069		0.263	0.793	-0.107		0.114	0.348
**Education**												
Education_MiddleSchool/Technical/HighSchool		References				References				References		
Education_NoSchool/ElementarySchool	0.109		0.273	0.689	0.248		0.371	0.504	0.073		0.163	0.656
Education_Bachelors/Masters/PhD	-0.044		0.273	0.871	-0.412		0.345	0.233	0.111		0.148	0.454
**Occupation**												
Occupation_Employed/Selfemployed		References				References				References		
Occupation_Unemployed	0.103		0.202	0.612	-0.051		0.213	0.811	0.010		0.109	0.923
Occupation_Homemaker	0.201		0.229	0.379	-0.058		0.289	0.842	0.033		0.129	0.799
Occupation_School/Apprenticeship/University	0.343		0.335	0.307	0.286		0.289	0.323	-0.023		0.157	0.886
Occupation_Other	0.198		0.212	0.351	0.025		0.219	0.909	0.079		0.104	0.447
Age	-0.040	**	0.018	0.028	-0.036		0.023	0.112	-0.005		0.013	0.702
Children	0.200		0.263	0.447	0.383		0.331	0.248	0.041		0.123	0.741
Income (ln)	0.264	***	0.082	0.001	0.230	**	0.093	0.014	0.091	**	0.043	0.035
Overall R^2	0.684				0.671				0.742			
Within R^2	0.173				n.a.				0.163			
Observations	2,970				2,970				10,755			

Note: * $p < 0.1$; ** $p < 0.05$; *** $p < 0.01$; For the analysis, we used different panel fixed effects estimations with robust standard errors in Stata 16.

(cont'd) Table S6. Total Market Model (robustness check)

Variable	Music consumption											
	Balanced fixed effects (model 1)				*Weighted fixed effects (model 2)*				*Unbalanced fixed effects (model 3)*			
	Coefficient		*SE*	*p-Value*	*Coefficient*		*SE*	*p-Value*	*Coefficient*		*SE*	*p-Value*
COVID-19	-0.143	***	0.027	0.000	-0.155	***	0.029	0.000	-0.075	***	0.018	0.000
Summer	0.004		0.021	0.859	0.004		0.025	0.857	0.001		0.012	0.943
Music related control variables												
MusicEducation	0.045		0.046	0.330	0.063		0.050	0.209	0.064	**	0.026	0.014
MusicAppreciation	0.023		0.014	0.105	0.019		0.015	0.211	0.031	***	0.008	0.000
ActiveListening	-0.021		0.013	0.116	-0.030	**	0.014	0.036	-0.022	***	0.007	0.003
MainstreamMusic	-0.018		0.016	0.273	-0.003		0.019	0.860	-0.008		0.009	0.345
Demographics												
**Marital status**												
MaritalStat_LivTogether_Partner		References				References				References		
MaritalStat_LivAlone	0.144		0.128	0.264	0.317	**	0.137	0.021	0.025		0.075	0.743
MaritalStat_LivAlone_Partner	-0.061		0.105	0.564	0.007		0.115	0.950	-0.041		0.067	0.543
**Education**												
Education_MiddleSchool/Technical/HighSchool		References				References				References		
Education_NoSchool/ElementarySchool	-0.020		0.183	0.915	0.146		0.265	0.582	-0.124		0.097	0.201
Education_Bachelors/Masters/PhD	-0.023		0.072	0.745	-0.066		0.108	0.537	0.157	***	0.060	0.009
**Occupation**												
Occupation_Employed/Selfemployed		References				References				References		
Occupation_Unemployed	-0.018		0.108	0.868	-0.230	*	0.122	0.060	-0.096		0.059	0.101
Occupation_Homemaker	-0.075		0.113	0.509	-0.251	**	0.118	0.034	-0.107		0.068	0.114
Occupation_School/Apprenticeship/University	-0.109		0.185	0.556	-0.289		0.237	0.223	-0.073		0.080	0.362
Occupation_Other	0.008		0.105	0.939	-0.169		0.119	0.155	-0.118	**	0.056	0.034
Age	-0.014		0.011	0.189	-0.006		0.010	0.579	-0.028	***	0.008	0.000
Children	-0.021		0.130	0.875	-0.094		0.144	0.516	0.014		0.069	0.839
Income (ln)	0.015		0.040	0.714	0.013		0.043	0.758	-0.001		0.021	0.969
Overall R^2	0.752				0.743				0.797			
Within R^2	0.031				n.a.				0.021			
Observations	2,960				2,960				10,704			

Note: * $p < 0.1$; **$p < 0.05$; ***$p < 0.01$; For the analysis, we used different panel fixed effects estimations with robust standard errors in Stata 16.

Table S7. Live Market Model (robustness check)

Variable	*Consumer spending*											
	Balanced fixed effects (model 1)				*Weighted fixed effects (model 2)*				*Unbalanced fixed effects (model 3)*			
	Coefficient		*SE*	*p-Value*	*Coefficient*		*SE*	*p-Value*	*Coefficient*		*SE*	*p-Value*
COVID-19	-0.630	***	0.061	0.000	-0.637	***	0.054	0.000	-0.566	***	0.035	0.000
Summer	0.146	***	0.040	0.000	0.144	***	0.050	0.004	0.111	***	0.024	0.000
Music related control variables												
MusicEducation	0.263	***	0.092	0.004	0.281	***	0.098	0.004	0.217	***	0.055	0.000
MusicAppreciation	-0.031		0.026	0.234	-0.027		0.027	0.333	0.003		0.015	0.840
ActiveListening	0.009		0.021	0.665	0.019		0.023	0.415	0.007		0.013	0.611
MainstreamMusic	0.048	*	0.029	0.095	0.059	*	0.033	0.075	0.019		0.015	0.213
**Purchase reason**												
PurchaseReason_Atmosphere	1.489	***	0.152	0.000	1.527	***	0.159	0.000	1.673	***	0.087	0.000
PurchaseReason_Flexibility	-0.025		0.113	0.827	0.136		0.147	0.357	0.061		0.067	0.364
PurchaseReason_Habit	0.220	**	0.088	0.013	0.328	***	0.100	0.001	0.226	***	0.049	0.000
PurchaseReason_SoundQuality	0.337	***	0.113	0.003	0.401	***	0.135	0.003	0.404	***	0.070	0.000
PurchaseReason_Mobility	-0.067		0.193	0.728	-0.008		0.203	0.970	0.264	**	0.106	0.013
PurchaseReason_Other	0.002		0.079	0.983	0.038		0.087	0.666	0.139	***	0.047	0.003
Demographics												
**Marital status**												
MaritalStat_LivTogether_Partner	References				References				References			
MaritalStat_LivAlone	0.048		0.267	0.856	0.015		0.237	0.950	0.067		0.124	0.592
MaritalStat_LivAlone_Partner	0.114		0.235	0.628	0.170		0.235	0.470	0.027		0.113	0.813
**Education**												
Education_MiddleSchool/Technical/HighSchool	References				References				References			
Education_NoSchool/ElementarySchool	-0.008		0.251	0.973	0.220		0.423	0.603	-0.102		0.135	0.450
Education_Bachelors/Masters/PhD	-0.073		0.229	0.751	-0.381		0.294	0.195	-0.066		0.148	0.656
**Occupation**												
Occupation_Employed/Selfemployed	References				References				References			
Occupation_Unemployed	0.152		0.161	0.345	-0.008		0.193	0.967	0.066		0.088	0.454
Occupation_Homemaker	0.220		0.184	0.232	0.040		0.228	0.859	0.027		0.112	0.810
Occupation_School/Apprenticeship/University	0.427		0.269	0.113	0.472	*	0.263	0.073	0.075		0.144	0.604
Occupation_Other	0.208		0.160	0.196	0.074		0.183	0.685	0.142		0.091	0.117
Age	-0.009		0.029	0.763	0.013		0.023	0.558	0.004		0.015	0.782
Children	0.342	*	0.192	0.075	0.351		0.285	0.219	0.103		0.112	0.359
Income (ln)	0.121	*	0.070	0.082	0.109		0.079	0.166	0.033		0.038	0.379
Overall R^2	0.503				0.500				0.623			
Within R^2	0.206				n.a.				0.189			
Observations	2,970				2,970				10,755			

Note: * $p < 0.1$; ** $p < 0.05$; *** $p < 0.01$; For the analysis, we used different panel fixed effects estimations with robust standard errors in Stata 16.

(cont'd) Table S7. Live Market Model (robustness check)

Variable	*Music consumption*											
	Balanced fixed effects (model 1)				*Weighted fixed effects (model 2)*				*Unbalanced fixed effects (model 3)*			
	Coefficient		*SE*	*p-Value*	*Coefficient*		*SE*	*p-Value*	*Coefficient*		*SE*	*p-Value*
COVID-19	-0.143	***	0.018	0.000	-0.145	***	0.018	0.000	-0.131	***	0.010	0.000
Summer	0.058	***	0.013	0.000	0.065	***	0.016	0.000	0.058	***	0.008	0.000
Music related control variables												
MusicEducation	-0.018		0.032	0.584	-0.044		0.033	0.175	0.003		0.018	0.864
MusicAppreciation	0.006		0.007	0.379	0.008		0.007	0.265	0.006		0.004	0.199
ActiveListening	0.004		0.007	0.624	0.000		0.008	0.985	0.009	**	0.004	0.035
MainstreamMusic	-0.007		0.008	0.364	-0.002		0.008	0.824	-0.008	*	0.004	0.064
Demographics												
**Marital status**												
MaritalStat_LivTogether_Partner			References				References				References	
MaritalStat_LivAlone	-0.005		0.082	0.952	0.031		0.088	0.724	0.011		0.038	0.769
MaritalStat_LivAlone_Partner	-0.012		0.066	0.851	0.043		0.075	0.565	-0.011		0.035	0.746
**Education**												
Education_MiddleSchool/Technical/HighSchool			References				References				References	
Education_NoSchool/ElementarySchool	0.024		0.118	0.837	0.233		0.242	0.336	-0.002		0.049	0.971
Education_Bachelors/Masters/PhD	-0.002		0.067	0.979	-0.049		0.092	0.597	0.040		0.044	0.369
**Occupation**												
Occupation_Employed/Selfemployed			References				References				References	
Occupation_Unemployed	0.031		0.040	0.431	0.029		0.042	0.490	-0.014		0.026	0.589
Occupation_Homemaker	0.067		0.051	0.189	0.041		0.062	0.507	0.031		0.031	0.313
Occupation_School/Apprenticeship/University	0.095		0.126	0.453	0.069		0.096	0.476	0.036		0.046	0.441
Occupation_Other	0.016		0.049	0.751	0.002		0.049	0.969	-0.013		0.028	0.632
Age	-0.002		0.008	0.842	0.003		0.008	0.669	-0.002		0.004	0.600
Children	-0.012		0.078	0.875	-0.056		0.089	0.527	0.003		0.040	0.946
Income (ln)	-0.015		0.024	0.545	-0.012		0.036	0.740	0.002		0.010	0.835
Overall R^2	0.429				0.458				0.548			
Within R^2	0.056				n.a.				0.046			
Observations	2,960				2,960				10,704			

Note: * $p < 0.1$; **$p < 0.05$; ***$p < 0.01$; For the analysis, we used different panel fixed effects estimations with robust standard errors in Stata

Table S8. Recorded Market Model (robustness check)

Variable	Consumer spending								
	Balanced fixed effects (model 1)			*Weighted fixed effects (model 2)*			*Unbalanced fixed effects (model 3)*		
	Coefficient	*SE*	*p-Value*	*Coefficient*	*SE*	*p-Value*	*Coefficient*	*SE*	*p-Value*
COVID-19	-0.149 ***	0.041	0.000	-0.140 ***	0.047	0.003	-0.133 ***	0.028	0.000
Summer	-0.037	0.036	0.308	-0.013	0.040	0.738	-0.061 ***	0.021	0.005
Music related control variables									
MusicEducation	0.120	0.074	0.104	0.176 **	0.079	0.026	0.119 ***	0.044	0.007
MusicAppreciation	0.053 **	0.023	0.018	0.051 **	0.023	0.024	0.066 ***	0.013	0.000
ActiveListening	-0.003	0.022	0.875	0.004	0.022	0.862	-0.003	0.012	0.799
MainstreamMusic	0.021	0.023	0.363	0.011	0.024	0.651	0.014	0.013	0.293
**Purchase reason**									
PurchaseReason_Atmosphere	0.306 ***	0.103	0.003	0.256 **	0.115	0.026	0.439 ***	0.060	0.000
PurchaseReason_Flexibility	0.712 ***	0.110	0.000	0.715 ***	0.107	0.000	0.670 ***	0.067	0.000
PurchaseReason_Habit	0.374 ***	0.079	0.000	0.350 ***	0.078	0.000	0.489 ***	0.049	0.000
PurchaseReason_SoundQuality	0.670 ***	0.110	0.000	0.703 ***	0.113	0.000	0.824 ***	0.066	0.000
PurchaseReason_Mobility	0.841 ***	0.192	0.000	0.788 ***	0.197	0.000	0.709 ***	0.103	0.000
PurchaseReason_Other	0.050	0.069	0.470	0.047	0.070	0.502	0.085 *	0.044	0.052
Demographics									
**Marital status**									
MaritalStat_LivTogether_Partner	References			References			References		
MaritalStat_LivAlone	-0.136	0.215	0.529	-0.024	0.235	0.917	-0.188 *	0.103	0.069
MaritalStat_LivAlone_Partner	-0.154	0.204	0.449	-0.069	0.200	0.731	-0.185 *	0.097	0.056
**Education**									
Education_MiddleSchool/Technical/HighSchool	References			References			References		
Education_NoSchool/ElementarySchool	0.004	0.190	0.983	0.089	0.178	0.618	0.103	0.135	0.445
Education_Bachelors/Masters/PhD	-0.048	0.214	0.823	-0.227	0.248	0.360	0.217 *	0.115	0.058
**Occupation**									
Occupation_Employed/Selfemployed	References			References			References		
Occupation_Unemployed	-0.019	0.163	0.907	-0.066	0.159	0.680	-0.025	0.092	0.788
Occupation_Homemaker	-0.001	0.175	0.994	-0.170	0.234	0.469	-0.026	0.109	0.809
Occupation_School/Apprenticeship/University	0.074	0.218	0.735	0.109	0.198	0.581	-0.066	0.123	0.591
Occupation_Other	0.022	0.155	0.885	-0.070	0.163	0.668	-0.021	0.084	0.808
Age	-0.037 ***	0.014	0.006	-0.039 **	0.019	0.036	-0.013	0.012	0.274
Children	-0.033	0.227	0.883	0.159	0.251	0.526	0.026	0.099	0.791
Income (ln)	0.198 ***	0.072	0.006	0.163 **	0.076	0.033	0.083 **	0.035	0.019
Overall R^2	0.720			0.713			0.759		
Within R^2	0.069			n.a.			0.066		
Observations	2,970			2,970			10,755		

Note: * p < 0.1; **p < 0.05; ***p < 0.01; For the analysis, we used different panel fixed effects estimations with robust standard errors in Stata 16.

(cont'd) Table S8. Recorded Market Model (robustness check)

Variable	Music consumption											
	Balanced fixed effects (model 1)				*Weighted fixed effects (model 2)*				*Unbalanced fixed effects (model 3)*			
	Coefficient		*SE*	*p-Value*	*Coefficient*		*SE*	*p-Value*	*Coefficient*		*SE*	*p-Value*
COVID-19	-0.123	***	0.027	0.000	-0.134	***	0.028	0.000	-0.055	***	0.018	0.003
Summer	-0.005		0.021	0.821	-0.005		0.025	0.844	-0.009		0.012	0.485
Music related control variables												
MusicEducation	0.048		0.045	0.287	0.072		0.049	0.143	0.064	**	0.026	0.014
MusicAppreciation	0.024		0.015	0.106	0.019		0.015	0.210	0.031	***	0.008	0.000
ActiveListening	-0.023	*	0.013	0.074	-0.032	**	0.014	0.025	-0.025	***	0.007	0.001
MainstreamMusic	-0.019		0.016	0.235	-0.006		0.019	0.746	-0.007		0.009	0.414
Demographics												
**Marital status**												
MaritalStat_LivTogether_Partner			References				References				References	
MaritalStat_LivAlone	0.153		0.129	0.234	0.319	**	0.134	0.018	0.019		0.076	0.807
MaritalStat_LivAlone_Partner	-0.059		0.107	0.585	0.010		0.115	0.928	-0.045		0.067	0.505
**Education**												
Education_MiddleSchool/Technical/HighSchool			References				References				References	
Education_NoSchool/ElementarySchool	-0.025		0.175	0.886	0.112		0.240	0.639	-0.119		0.096	0.215
Education_Bachelors/Masters/PhD	-0.033		0.070	0.636	-0.072		0.104	0.490	0.145	**	0.060	0.015
**Occupation**												
Occupation_Employed/Selfemployed			References				References				References	
Occupation_Unemployed	-0.016		0.109	0.884	-0.230	*	0.122	0.059	-0.090		0.059	0.126
Occupation_Homemaker	-0.088		0.113	0.440	-0.260	**	0.116	0.025	-0.113	*	0.068	0.098
Occupation_School/Apprenticeship/University	-0.150		0.170	0.380	-0.311		0.231	0.178	-0.091		0.079	0.253
Occupation_Other	0.004		0.106	0.971	-0.174		0.119	0.145	-0.114	**	0.056	0.042
Age	-0.013		0.010	0.180	-0.006		0.010	0.572	-0.029	***	0.008	0.000
Children	-0.017		0.130	0.896	-0.085		0.143	0.550	0.012		0.068	0.855
Income (ln)	0.022		0.039	0.582	0.018		0.041	0.665	0.002		0.021	0.914
Overall R^2	0.756				0.713				0.798			
Within R^2	0.027				n.a.				0.018			
Observations	2,960				2,960				10,704			

Note: * p < 0.1; **p < 0.05; ***p < 0.01; For the analysis, we used different panel fixed effects estimations with robust standard errors in Stata 16.

Table S9. Recorded Submarket Model (robustness check)

Variable	Consumer spending on premium streaming											
	Balanced fractional multinomial logit (model 1)				*Weighted fractional multinomial logit (model 2)*				*Unbalanced fractional multinomial logit (model 3)*			
	APE		*SE*	*p-Value*	*APE*		*SE*	*p-Value*	*APE*		*SE*	*p-Value*
COVID-19	0.042	***	0.010	0.000	0.037	***	0.013	0.005	0.037	***	0.006	0.000
Summer	-0.006		0.008	0.481	-0.000		0.010	0.995	-0.004		0.005	0.357
Music related control variables												
MusicEducation	0.018		0.014	0.196	0.011		0.017	0.504	0.006		0.006	0.294
MusicAppreciation	0.011	**	0.005	0.034	0.015	**	0.007	0.026	0.012	***	0.002	0.000
ActiveListening	-0.016	***	0.005	0.003	-0.018	***	0.007	0.007	-0.012	***	0.003	0.000
MainstreamMusic	0.019	***	0.006	0.003	0.024	***	0.008	0.002	0.006	**	0.003	0.025
**Purchase reason**												
PurchaseReason_Atmosphere	-0.165	***	0.034	0.000	-0.209	***	0.042	0.000	-0.102	***	0.015	0.000
PurchaseReason_Flexibility	0.003		0.028	0.915	-0.019		0.035	0.592	0.027	**	0.013	0.047
PurchaseReason_Habit	-0.151	***	0.028	0.000	-0.179	***	0.035	0.000	-0.098	***	0.012	0.000
PurchaseReason_SoundQuality	-0.145	***	0.030	0.000	-0.136	***	0.037	0.000	-0.104	***	0.014	0.000
PurchaseReason_Mobility	0.027		0.039	0.490	-0.000		0.046	0.993	0.052	***	0.018	0.004
PurchaseReason_Other	-0.180	***	0.030	0.000	-0.217	***	0.036	0.000	-0.130	***	0.014	0.000
Demographics												
**Marital status**												
MaritalStat_LivTogether_Partner	References				References				References			
MaritalStat_LivAlone	-0.011		0.024	0.664	0.008		0.032	0.804	-0.019	*	0.011	0.083
MaritalStat_LivAlone_Partner	-0.021		0.037	0.569	-0.002		0.043	0.961	0.016		0.016	0.298
**Education**												
Education_MiddleSchool/Technical/HighSchool	References				References				References			
Education_NoSchool/ElementarySchool	0.021		0.039	0.592	0.023		0.045	0.601	-0.013		0.016	0.427
Education_Bachelors/Masters/PhD	-0.005		0.023	0.836	-0.008		0.028	0.777	-0.002		0.011	0.820
**Occupation**												
Occupation_Employed/Selfemployed	References				References				References			
Occupation_Unemployed	-0.056		0.034	0.105	-0.033		0.050	0.508	-0.020		0.019	0.306
Occupation_Homemaker	-0.023		0.033	0.492	-0.014		0.040	0.724	-0.007		0.020	0.713
Occupation_School/Apprenticeship/University	0.051		0.059	0.389	0.042		0.062	0.504	0.050	***	0.019	0.008
Occupation_Other	0.011		0.038	0.771	0.029		0.046	0.526	-0.008		0.017	0.643
GenderFemale	-0.057	***	0.022	0.010	-0.051	*	0.026	0.054	-0.041	***	0.010	0.000
Age	-0.004	***	0.001	0.000	-0.005	***	0.001	0.000	-0.005	***	0.000	0.000
Children	-0.002		0.023	0.946	-0.007		0.027	0.798	0.007		0.011	0.545
Income (ln)	0.025		0.017	0.148	0.040	*	0.022	0.067	0.022	***	0.007	0.002
Wald chi^2	732.30				667.38				2221.50			
Prob > chi^2	0.000				0.000				0.000			
Observations	2,970				2,970				10,755			

Note: * p < 0.1; **p < 0.05; ***p < 0.01; For the analysis, we used a fractional multinomial logit model and report average partial effects with cluster-robust standard errors with respect to participants. The fractional multinomial logit model is estimated with the STATA 16 and the module FMLOGIT [42]

(cont'd) Table S9. Recorded Submarket Model (robustness check)

Variable	Consumer spending on physical								
	Balanced fractional multinomial logit (model 1)			*Weighted fractional multinomial logit (model 2)*			*Unbalanced fractional multinomial logit (model 3)*		
	APE	*SE*	*p-Value*	*APE*	*SE*	*p-Value*	*APE*	*SE*	*p-Value*
COVID-19	-0.074 ***	0.011	0.000	-0.066 ***	0.011	0.000	-0.055 ***	0.007	0.000
Summer	0.004	0.011	0.703	0.006	0.011	0.599	-0.005	0.006	0.405
Music related control variables									
MusicEducation	0.012	0.011	0.265	0.008	0.012	0.514	0.016 ***	0.005	0.001
MusicAppreciation	0.047 ***	0.006	0.000	0.043 ***	0.007	0.000	0.035 ***	0.003	0.000
ActiveListening	0.002	0.005	0.732	0.006	0.006	0.368	0.007 ***	0.002	0.004
MainstreamMusic	-0.002	0.006	0.773	-0.004	0.006	0.533	-0.006 **	0.003	0.014
**Purchase reason**									
PurchaseReason_Atmosphere	0.317 ***	0.058	0.000	0.304 ***	0.061	0.000	0.391 ***	0.027	0.000
PurchaseReason_Flexibility	0.296 ***	0.059	0.000	0.291 ***	0.063	0.000	0.347 ***	0.028	0.000
PurchaseReason_Habit	0.292 ***	0.057	0.000	0.277 ***	0.061	0.000	0.367 ***	0.027	0.000
PurchaseReason_SoundQuality	0.341 ***	0.058	0.000	0.324 ***	0.061	0.000	0.455 ***	0.027	0.000
PurchaseReason_Mobility	0.286 ***	0.067	0.000	0.281 ***	0.070	0.000	0.339 ***	0.032	0.000
PurchaseReason_Other	0.213 ***	0.058	0.000	0.225 ***	0.062	0.000	0.292 ***	0.028	0.000
Demographics									
**Marital status**									
MaritalStat_LivTogether_Partner		References			References			References	
MaritalStat_LivAlone	-0.009	0.021	0.671	-0.005	0.022	0.814	-0.008	0.010	0.413
MaritalStat_LivAlone_Partner	0.035	0.038	0.354	0.015	0.036	0.678	0.014	0.016	0.392
**Education**									
Education_MiddleSchool/Technical/HighSchool		References			References			References	
Education_NoSchool/ElementarySchool	-0.013	0.033	0.699	-0.027	0.033	0.406	-0.011	0.015	0.474
Education_Bachelors/Masters/PhD	0.017	0.021	0.410	-0.012	0.021	0.591	-0.010	0.009	0.299
**Occupation**									
Occupation_Employed/Selfemployed		References			References			References	
Occupation_Unemployed	0.020	0.042	0.627	0.004	0.038	0.920	-0.017	0.017	0.319
Occupation_Homemaker	-0.009	0.033	0.775	-0.016	0.033	0.627	-0.014	0.018	0.427
Occupation_School/Apprenticeship/University	-0.053	0.054	0.333	-0.016	0.054	0.769	-0.014	0.021	0.494
Occupation_Other	-0.012	0.029	0.670	-0.014	0.028	0.609	-0.008	0.013	0.539
GenderFemale	-0.031	0.019	0.109	-0.040 **	0.020	0.046	-0.007	0.009	0.400
Age	0.002 *	0.001	0.058	0.001	0.001	0.350	0.002 ***	0.000	0.000
Children	0.026	0.020	0.200	0.033	0.021	0.117	0.013	0.009	0.176
Income (ln)	-0.022	0.015	0.131	-0.041 ***	0.015	0.006	-0.004	0.007	0.573
Wald chi^2	732.30			667.38			2221.50		
Prob > chi^2	0.000			0.000			0.000		
Observations	2,970			2,970			10,755		

Note: * $p < 0.1$; ** $p < 0.05$; *** $p < 0.01$; For the analysis, we used a fractional multinomial logit model and report average partial effects with cluster-robust standard errors with respect to participants. The fractional multinomial logit model is estimated with the STATA 16 and the module FMLOGIT [42]

(cont'd) Table S9. Recorded Submarket Model (robustness check)

	Consumer spending on digital downloads								
Variable	*Balanced fractional multinomial logit (model 1)*			*Weighted fractional multinomial logit (model 2)*			*Unbalanced fractional multinomial logit (model 3)*		
	APE	*SE*	*p-Value*	*APE*	*SE*	*p-Value*	*APE*	*SE*	*p-Value*
COVID-19	-0.011 *	0.006	0.058	-0.017 **	0.007	0.015	-0.009 **	0.004	0.025
Summer	-0.002	0.006	0.758	-0.001	0.007	0.920	-0.000	0.003	0.923
Music related control variables									
MusicEducation	0.002	0.007	0.818	-0.002	0.008	0.828	0.002	0.003	0.565
MusicAppreciation	0.011 ***	0.004	0.005	0.012 ***	0.005	0.009	0.009 ***	0.002	0.000
ActiveListening	0.002	0.004	0.519	0.001	0.004	0.836	0.000	0.002	0.892
MainstreamMusic	-0.001	0.004	0.695	-0.003	0.005	0.576	0.005 ***	0.002	0.005
**Purchase reason**									
PurchaseReason_Atmosphere	0.214 ***	0.056	0.000	0.256 ***	0.062	0.000	0.050 ***	0.015	0.001
PurchaseReason_Flexibility	0.336 ***	0.058	0.000	0.363 ***	0.061	0.000	0.143 ***	0.015	0.000
PurchaseReason_Habit	0.278 ***	0.057	0.000	0.306 ***	0.061	0.000	0.090 ***	0.014	0.000
PurchaseReason_SoundQuality	0.285 ***	0.057	0.000	0.314 ***	0.061	0.000	0.089 ***	0.014	0.000
PurchaseReason_Mobility	0.315 ***	0.061	0.000	0.338 ***	0.065	0.000	0.149 ***	0.016	0.000
PurchaseReason_Other	0.259 ***	0.058	0.000	0.279 ***	0.060	0.000	0.058 ***	0.015	0.000
Demographics									
**Marital status**									
MaritalStat_LivTogether_Partner	References			References			References		
MaritalStat_LivAlone	0.020	0.014	0.152	0.024	0.015	0.114	0.006	0.006	0.290
MaritalStat_LivAlone_Partner	-0.013	0.022	0.548	-0.021	0.020	0.309	0.001	0.010	0.951
**Education**									
Education_MiddleSchool/Technical/HighSchool	References			References			References		
Education_NoSchool/ElementarySchool	0.015	0.029	0.594	0.015	0.030	0.617	0.003	0.011	0.753
Education_Bachelors/Masters/PhD	-0.011	0.012	0.352	-0.002	0.015	0.887	-0.000	0.006	0.991
**Occupation**									
Occupation_Employed/Selfemployed	References			References			References		
Occupation_Unemployed	-0.034 **	0.016	0.034	-0.021	0.021	0.325	-0.016	0.011	0.140
Occupation_Homemaker	-0.031 *	0.017	0.070	-0.032 *	0.017	0.069	-0.022 **	0.010	0.024
Occupation_School/Apprenticeship/University	-0.005	0.030	0.856	-0.002	0.035	0.949	-0.011	0.009	0.212
Occupation_Other	-0.012	0.025	0.640	-0.014	0.025	0.574	0.001	0.012	0.961
GenderFemale	0.003	0.013	0.804	0.005	0.014	0.737	-0.004	0.006	0.424
Age	-0.001	0.001	0.128	-0.001	0.001	0.379	-0.001 ***	0.000	0.000
Children	-0.006	0.012	0.628	-0.004	0.013	0.780	-0.009	0.006	0.128
Income (ln)	0.016 *	0.009	0.065	0.017 *	0.009	0.052	0.014 ***	0.004	0.001
Wald chi^2	732.30			667.38			2221.50		
Prob > chi^2	0.000			0.000			0.000		
Observations	2,970			2,970			10,755		

Note: * p < 0.1; **p < 0.05; ***p < 0.01; For the analysis, we used a fractional multinomial logit model and report average partial effects with cluster-robust standard errors with respect to participants. The fractional multinomial logit model is estimated with the STATA 16 and the module FMLOGIT [42]

(cont'd) Table S9. Recorded Submarket Model (robustness check)

Variable	No consumer spending											
	Balanced fractional multinomial logit (model 1)				*Weighted fractional multinomial logit (model 2)*				*Unbalanced fractional multinomial logit (model 3)*			
	APE		*SE*	*p-Value*	*APE*		*SE*	*p-Value*	*APE*		*SE*	*p-Value*
COVID-19	0.043	***	0.013	0.001	0.046	***	0.016	0.005	0.027	***	0.008	0.001
Summer	0.003		0.012	0.768	-0.005		0.014	0.725	0.010		0.007	0.177
Music related control variables												
MusicEducation	-0.032	*	0.016	0.052	-0.017		0.020	0.387	-0.024	***	0.007	0.001
MusicAppreciation	-0.069	***	0.006	0.000	-0.069	***	0.008	0.000	-0.057	***	0.003	0.000
ActiveListening	0.012	*	0.007	0.088	0.012		0.008	0.135	0.004		0.003	0.180
MainstreamMusic	-0.016	**	0.008	0.038	-0.018	**	0.009	0.044	-0.005		0.003	0.185
**Purchase reason**												
PurchaseReason_Atmosphere	-0.366	***	0.054	0.000	-0.351	***	0.058	0.000	-0.339	***	0.024	0.000
PurchaseReason_Flexibility	-0.635	***	0.050	0.000	-0.635	***	0.055	0.000	-0.517	***	0.024	0.000
PurchaseReason_Habit	-0.418	***	0.049	0.000	-0.404	***	0.053	0.000	-0.359	***	0.022	0.000
PurchaseReason_SoundQuality	-0.481	***	0.052	0.000	-0.502	***	0.055	0.000	-0.441	***	0.024	0.000
PurchaseReason_Mobility	-0.629	***	0.069	0.000	-0.618	***	0.075	0.000	-0.539	***	0.032	0.000
PurchaseReason_Other	-0.292	***	0.051	0.000	-0.287	***	0.055	0.000	-0.220	***	0.023	0.000
Demographics												
**Marital status**												
MaritalStat_LivTogether_Partner	References				References				References			
MaritalStat_LivAlone	-0.001		0.029	0.984	-0.027		0.035	0.439	0.021		0.013	0.120
MaritalStat_LivAlone_Partner	-0.002		0.043	0.968	0.008		0.048	0.869	-0.031		0.020	0.123
**Education**												
Education_MiddleSchool/Technical/HighSchool	References				References				References			
Education_NoSchool/ElementarySchool	-0.023		0.044	0.601	-0.011		0.049	0.821	0.020		0.019	0.291
Education_Bachelors/Masters/PhD	-0.001		0.028	0.971	0.022		0.033	0.507	0.012		0.013	0.351
**Occupation**												
Occupation_Employed/Selfemployed	References				References				References			
Occupation_Unemployed	0.070		0.045	0.120	0.051		0.047	0.285	0.052	**	0.022	0.017
Occupation_Homemaker	0.062		0.042	0.138	0.062		0.044	0.161	0.043	*	0.023	0.066
Occupation_School/Apprenticeship/University	0.006		0.072	0.931	-0.024		0.071	0.737	-0.025		0.025	0.310
Occupation_Other	0.014		0.044	0.754	-0.001		0.048	0.988	0.016		0.019	0.416
GenderFemale	0.084	***	0.026	0.001	0.085	***	0.029	0.003	0.053	***	0.012	0.000
Age	0.003	***	0.001	0.007	0.004	***	0.001	0.001	0.004	***	0.001	0.000
Children	-0.019		0.027	0.493	-0.023		0.030	0.453	-0.011		0.013	0.407
Income (ln)	-0.019		0.020	0.346	-0.016		0.022	0.471	-0.032	***	0.009	0.000
Wald chi^2	732.30				667.38				2221.50			
Prob > chi^2	0.000				0.000				0.000			
Observations	2,970				2,970				10,755			

Note: * p < 0.1; **p < 0.05; ***p < 0.01; For the analysis, we used a fractional multinomial logit model and report average partial effects with cluster-robust standard errors with respect to participants. The fractional multinomial logit model is estimated with the STATA 16 and the module FMLOGIT [42]

(cont'd) Table S9. Recorded Submarket Model (robustness check)

Variable	*Music consumption – Premium streaming*								
	Balanced fractional multinomial logit (model 1)			*Weighted fractional multinomial logit (model 2)*			*Unbalanced fractional multinomial logit (model 3)*		
	APE	*SE*	*p-Value*	*APE*	*SE*	*p-Value*	*APE*	*SE*	*p-Value*
COVID-19	0.034 ***	0.005	0.000	0.042 ***	0.008	0.000	0.035 ***	0.004	0.000
Summer	-0.005	0.004	0.237	-0.006	0.006	0.337	-0.003	0.003	0.347
Music related control variables									
MusicEducation	0.006	0.008	0.416	0.009	0.012	0.473	0.002	0.004	0.491
MusicAppreciation	0.006 *	0.003	0.054	0.010 *	0.006	0.070	0.008 ***	0.002	0.000
ActiveListening	-0.010 ***	0.004	0.007	-0.015 ***	0.005	0.003	-0.006 ***	0.002	0.001
MainstreamMusic	0.005	0.004	0.241	0.011 **	0.005	0.045	0.001	0.002	0.640
Demographics									
**Marital status**									
MaritalStat_LivTogether_Partner	References			References			References		
MaritalStat_LivAlone	-0.001	0.016	0.956	0.008	0.022	0.706	-0.003	0.008	0.680
MaritalStat_LivAlone_Partner	0.004	0.028	0.878	-0.012	0.033	0.720	0.017	0.011	0.122
**Education**									
Education_MiddleSchool/Technical/HighSchool	References			References			References		
Education_NoSchool/ElementarySchool	0.052 *	0.031	0.090	0.090 **	0.044	0.041	0.006	0.012	0.599
Education_Bachelors/Masters/PhD	-0.008	0.014	0.578	-0.012	0.018	0.490	0.007	0.007	0.327
**Occupation**									
Occupation_Employed/Selfemployed	References			References			References		
Occupation_Unemployed	-0.052 ***	0.014	0.000	-0.059 ***	0.022	0.008	-0.021 *	0.011	0.054
Occupation_Homemaker	0.019	0.026	0.479	0.034	0.035	0.330	0.006	0.014	0.654
Occupation_School/Apprenticeship/University	0.099 *	0.057	0.081	0.091	0.059	0.124	0.046 ***	0.014	0.001
Occupation_Other	-0.001	0.029	0.973	-0.026	0.030	0.374	-0.001	0.014	0.943
GenderFemale	-0.032 **	0.015	0.038	-0.028	0.021	0.181	-0.019 ***	0.006	0.004
Age	-0.004 ***	0.001	0.000	-0.004 ***	0.001	0.000	-0.005 ***	0.000	0.000
Children	0.009	0.014	0.536	0.000	0.018	0.985	0.007	0.007	0.300
Income (ln)	0.015	0.010	0.127	0.026 *	0.015	0.082	0.014 ***	0.004	0.001
Wald chi^2	765.37			789.85			2452.33		
Prob > chi^2	0.000			0.000			0.000		
Observations	2,960			2,960			10,704		

Note: * $p < 0.1$; **$p < 0.05$; ***$p < 0.01$; For the analysis, we used a fractional multinomial logit model and report average partial effects with cluster-robust standard errors with respect to participants. The fractional multinomial logit model is estimated with the STATA 16 and the module FMLOGIT [42]

(cont'd) Table S9. Recorded Submarket Model (robustness check)

Variable	*Music consumption – Free streaming*								
	Balanced fractional multinomial logit (model 1)			*Weighted fractional multinomial logit (model 2)*			*Unbalanced fractional multinomial logit (model 3)*		
	APE	*SE*	*p-Value*	*APE*	*SE*	*p-Value*	*APE*	*SE*	*p-Value*
COVID-19	0.007	0.004	0.101	0.007	0.008	0.336	0.003	0.003	0.298
Summer	-0.002	0.003	0.549	0.002	0.006	0.669	0.002	0.002	0.412
Music related control variables									
MusicEducation	0.004	0.006	0.488	0.005	0.009	0.606	0.001	0.002	0.657
MusicAppreciation	-0.007 ***	0.002	0.000	-0.011 ***	0.003	0.000	-0.005 ***	0.001	0.000
ActiveListening	0.001	0.002	0.736	0.001	0.004	0.789	0.002 **	0.001	0.024
MainstreamMusic	-0.001	0.002	0.548	-0.005	0.004	0.165	-0.001	0.001	0.646
Demographics									
**Marital status**									
MaritalStat_LivTogether_Partner		References			References			References	
MaritalStat_LivAlone	0.018 *	0.011	0.085	0.023	0.016	0.145	0.011 **	0.005	0.020
MaritalStat_LivAlone_Partner	-0.002	0.013	0.879	0.002	0.022	0.941	0.002	0.006	0.710
**Education**									
Education_MiddleSchool/Technical/HighSchool		References			References			References	
Education_NoSchool/ElementarySchool	0.006	0.014	0.649	-0.001	0.019	0.965	-0.011 **	0.005	0.047
Education_Bachelors/Masters/PhD	0.016	0.011	0.136	0.012	0.015	0.428	0.005	0.005	0.294
**Occupation**									
Occupation_Employed/Selfemployed		References			References			References	
Occupation_Unemployed	0.009	0.016	0.586	0.012	0.022	0.580	0.017 *	0.009	0.054
Occupation_Homemaker	-0.012	0.013	0.377	-0.017	0.016	0.288	-0.006	0.007	0.438
Occupation_School/Apprenticeship/University	0.032	0.025	0.194	0.024	0.024	0.302	0.026 ***	0.010	0.009
Occupation_Other	0.013	0.015	0.388	0.020	0.029	0.488	0.002	0.007	0.772
GenderFemale	-0.010	0.009	0.275	-0.019	0.013	0.166	-0.006	0.004	0.126
Age	-0.002 ***	0.000	0.001	-0.002 ***	0.001	0.001	-0.001 ***	0.000	0.000
Children	-0.015	0.010	0.112	-0.021	0.014	0.135	-0.008 *	0.004	0.080
Income (ln)	0.002	0.004	0.690	0.001	0.006	0.862	-0.001	0.002	0.538
Wald chi^2	765.37			789.85			2452.33		
Prob > chi^2	0.000			0.000			0.000		
Observations	2,960			2,960			10,704		

Note: * $p < 0.1$; **$p < 0.05$; ***$p < 0.01$; For the analysis, we used a fractional multinomial logit model and report average partial effects with cluster-robust standard errors with respect to participants. The fractional multinomial logit model is estimated with the STATA 16 and the module FMLOGIT [42]

(cont'd) Table S9. Recorded Submarket Model (robustness check)

	Music consumption – Physical								
Variable	*Balanced fractional multinomial logit (model 1)*			*Weighted fractional multinomial logit (model 2)*			*Unbalanced fractional multinomial logit (model 3)*		
	APE	*SE*	*p-Value*	*APE*	*SE*	*p-Value*	*APE*	*SE*	*p-Value*
COVID-19	-0.007	0.006	0.187	-0.005	0.003	0.116	-0.010 *	0.006	0.079
Summer	0.006	0.005	0.196	-0.004	0.003	0.149	0.005	0.005	0.315
Music related control variables									
MusicEducation	0.005	0.009	0.568	0.005	0.003	0.127	0.005	0.009	0.567
MusicAppreciation	0.031 ***	0.004	0.000	0.025 ***	0.002	0.000	0.024 ***	0.004	0.000
ActiveListening	0.010 ***	0.004	0.007	0.011 ***	0.001	0.000	0.009 ***	0.003	0.005
MainstreamMusic	-0.019 ***	0.004	0.000	-0.012 ***	0.002	0.000	-0.019 ***	0.004	0.000
Demographics									
**Marital status**									
MaritalStat_LivTogether_Partner		References			References			References	
MaritalStat_LivAlone	0.010	0.016	0.533	0.011 *	0.006	0.081	0.008	0.014	0.583
MaritalStat_LivAlone_Partner	0.011	0.024	0.639	0.022 **	0.011	0.037	0.023	0.026	0.379
**Education**									
Education_MiddleSchool/Technical/HighSchool		References			References			References	
Education_NoSchool/ElementarySchool	0.010	0.023	0.668	-0.008	0.008	0.362	0.008	0.023	0.734
Education_Bachelors/Masters/PhD	0.026	0.016	0.113	0.003	0.006	0.688	0.015	0.015	0.290
**Occupation**									
Occupation_Employed/Selfemployed		References			References			References	
Occupation_Unemployed	0.017	0.020	0.391	0.009	0.009	0.308	0.007	0.017	0.668
Occupation_Homemaker	0.011	0.026	0.675	0.008	0.012	0.534	0.011	0.025	0.666
Occupation_School/Apprenticeship/University	0.026	0.045	0.564	-0.008	0.013	0.562	0.049	0.043	0.262
Occupation_Other	-0.009	0.018	0.604	-0.005	0.008	0.537	-0.011	0.015	0.483
GenderFermale	0.030 **	0.014	0.031	0.013 **	0.006	0.024	0.026 **	0.013	0.046
Age	0.003 ***	0.001	0.000	0.002 ***	0.000	0.000	0.003 ***	0.001	0.000
Children	0.005	0.015	0.741	-0.002	0.006	0.696	0.014	0.014	0.341
Income (ln)	-0.008	0.009	0.359	-0.009 **	0.004	0.033	-0.008	0.008	0.311
Wald chi^2	765.37			789.85			2452.33		
Prob > chi^2	0.000			0.000			0.000		
Observations	2,960			2,960			10,704		

Note: * p < 0.1; **p < 0.05; ***p < 0.01; For the analysis, we used a fractional multinomial logit model and report average partial effects with cluster-robust standard errors with respect to participants. The fractional multinomial logit model is estimated with the STATA 16 and the module FMLOGIT [42]

(cont'd) Table S9. Recorded Submarket Model (robustness check)

Variable	*Music consumption – Digital downloads*								
	Balanced fractional multinomial logit (model 1)			*Weighted fractional multinomial logit (model 2)*			*Unbalanced fractional multinomial logit (model 3)*		
	APE	*SE*	*p-Value*	*APE*	*SE*	*p-Value*	*APE*	*SE*	*p-Value*
COVID-19	-0.011 *	0.006	0.076	-0.018 **	0.008	0.030	-0.014 ***	0.004	0.001
Summer	0.000	0.005	0.980	0.001	0.006	0.923	-0.000	0.004	0.921
Music related control variables									
MusicEducation	0.005	0.009	0.622	-0.001	0.011	0.964	0.004	0.004	0.409
MusicAppreciation	0.011 ***	0.004	0.003	0.015 ***	0.004	0.000	0.009 ***	0.002	0.000
ActiveListening	0.009 **	0.004	0.034	0.010 **	0.004	0.027	0.007 ***	0.002	0.000
MainstreamMusic	-0.021 ***	0.005	0.000	-0.024 ***	0.005	0.000	-0.014 ***	0.002	0.000
Demographics									
**Marital status**									
MaritalStat_LivTogether_Partner		References			References			References	
MaritalStat_LivAlone	-0.076 ***	0.026	0.003	0.021	0.020	0.281	0.031 ***	0.008	0.000
MaritalStat_LivAlone_Partner	-0.027	0.042	0.520	0.008	0.032	0.813	0.006	0.012	0.634
**Education**									
Education_MiddleSchool/Technical/HighSchool		References			References			References	
Education_NoSchool/ElementarySchool	-0.067 *	0.038	0.078	-0.051 **	0.021	0.016	-0.001	0.011	0.908
Education_Bachelors/Masters/PhD	-0.040 *	0.024	0.097	0.012	0.021	0.562	0.003	0.008	0.679
**Occupation**									
Occupation_Employed/Selfemployed		References			References			References	
Occupation_Unemployed	-0.022	0.039	0.578	0.085 **	0.040	0.036	0.036 **	0.015	0.016
Occupation_Homemaker	0.008	0.046	0.862	-0.023	0.031	0.452	-0.013	0.014	0.342
Occupation_School/Apprenticeship/University	-0.058	0.068	0.387	-0.059 **	0.027	0.030	0.017	0.013	0.205
Occupation_Other	-0.015	0.035	0.674	-0.009	0.028	0.736	0.014	0.012	0.236
GenderFemale	0.087 ***	0.023	0.000	-0.014	0.016	0.403	-0.018 ***	0.007	0.010
Age	0.005 ***	0.001	0.000	-0.003 ***	0.001	0.000	-0.003 ***	0.000	0.000
Children	0.019	0.024	0.429	-0.020	0.016	0.219	-0.011	0.007	0.134
Income (ln)	0.015	0.016	0.336	-0.007	0.013	0.583	-0.005	0.005	0.338
Wald chi^2	765.37			789.85			2452.33		
Prob > chi^2	0.000			0.000			0.000		
Observations	2,960			2,960			10,704		

Note: * p < 0.1; **p < 0.05; ***p < 0.01; For the analysis, we used a fractional multinomial logit model and report average partial effects with cluster-robust standard errors with respect to participants. The fractional multinomial logit model is estimated with the STATA 16 and the module FMLOGIT [42]

(cont'd) Table S9. Recorded Submarket Model (robustness check)

Variable	Music consumption – Radio											
	Balanced fractional multinomial logit (model 1)				*Weighted fractional multinomial logit (model 2)*				*Unbalanced fractional multinomial logit (model 3)*			
	APE		*SE*	*p-Value*	*APE*		*SE*	*p-Value*	*APE*		*SE*	*p-Value*
COVID-19	-0.049	***	0.010	0.000	-0.041	***	0.011	0.000	-0.043	***	0.006	0.000
Summer	0.013		0.008	0.113	0.008		0.008	0.332	0.009	*	0.005	0.060
Music related control variables												
MusicEducation	-0.009		0.014	0.542	-0.010		0.015	0.494	-0.012	*	0.006	0.064
MusicAppreciation	-0.028	***	0.005	0.000	-0.029	***	0.005	0.000	-0.028	***	0.003	0.000
ActiveListening	-0.013	**	0.006	0.032	-0.008		0.006	0.170	-0.016	***	0.003	0.000
MainstreamMusic	0.033	***	0.007	0.000	0.034	***	0.007	0.000	0.028	***	0.003	0.000
Demographics												
**Marital status**												
MaritalStat_LivTogether_Partner	References				References				References			
MaritalStat_LivAlone	-0.076	***	0.026	0.003	-0.064	**	0.027	0.018	-0.063	***	0.012	0.000
MaritalStat_LivAlone_Partner	-0.027		0.042	0.520	0.006		0.042	0.888	-0.034	*	0.018	0.063
**Education**												
Education_MiddleSchool/Technical/HighSchool	References				References				References			
Education_NoSchool/ElementarySchool	-0.067	*	0.038	0.078	-0.071	*	0.040	0.075	-0.001		0.017	0.936
Education_Bachelors/Masters/PhD	-0.040	*	0.024	0.097	-0.029		0.026	0.263	-0.016		0.011	0.151
**Occupation**												
Occupation_Employed/Selfemployed	References				References				References			
Occupation_Unemployed	-0.022		0.039	0.578	-0.034		0.042	0.421	-0.052	***	0.019	0.005
Occupation_Homemaker	0.008		0.046	0.862	0.022		0.048	0.642	-0.019		0.022	0.391
Occupation_School/Apprenticeship/University	-0.058		0.068	0.387	-0.049		0.061	0.426	-0.035	*	0.021	0.100
Occupation_Other	-0.015		0.035	0.674	0.013		0.053	0.806	-0.033	**	0.017	0.049
GenderFermale	0.087	***	0.023	0.000	0.075	***	0.024	0.002	0.051	***	0.011	0.000
Age	0.005	***	0.001	0.000	0.005	***	0.001	0.000	0.007	***	0.000	0.000
Children	0.019		0.024	0.429	0.025		0.024	0.300	0.016		0.011	0.146
Income (ln)	0.015		0.016	0.336	0.007		0.016	0.657	0.009		0.007	0.222
Wald chi^2	765.37				789.85				2452.33			
Prob > chi^2	0.000				0.000				0.000			
Observations	2,960				2,960				10,704			

Note: * $p < 0.1$; ** $p < 0.05$; *** $p < 0.01$; For the analysis, we used a fractional multinomial logit model and report average partial effects with cluster-robust standard errors with respect to participants. The fractional multinomial logit model is estimated with the STATA 16 and the module FMLOGIT [42]

(cont'd) Table S9. Recorded Submarket Model (robustness check)

Variable	*Music consumption – Online radio*											
	Balanced fractional multinomial logit (model 1)				*Weighted fractional multinomial logit (model 2)*				*Unbalanced fractional multinomial logit (model 3)*			
	APE		*SE*	*p-Value*	*APE*		*SE*	*p-Value*	*APE*		*SE*	*p-Value*
COVID-19	0.012	**	0.006	0.033	0.010	*	0.006	0.086	0.012	***	0.004	0.001
Summer	-0.008		0.005	0.127	-0.005		0.005	0.275	-0.004		0.003	0.228
Music related control variables												
MusicEducation	-0.014		0.010	0.151	-0.009		0.010	0.344	0.003		0.004	0.395
MusicAppreciation	-0.002		0.004	0.557	-0.001		0.004	0.693	-0.001		0.002	0.659
ActiveListening	-0.006	*	0.004	0.100	-0.004		0.003	0.192	-0.004	**	0.002	0.023
MainstreamMusic	0.008	*	0.004	0.069	0.008	**	0.004	0.046	0.003	*	0.002	0.072
Demographics												
**Marital status**												
MaritalStat_LivTogether_Partner			References				References				References	
MaritalStat_LivAlone	-0.012		0.017	0.484	-0.018		0.017	0.306	-0.010		0.007	0.157
MaritalStat_LivAlone_Partner	-0.015		0.026	0.548	-0.023		0.025	0.368	-0.009		0.011	0.415
**Education**												
Education_MiddleSchool /Technical/HighSchool			References				References				References	
Education_NoSchool/ElementarySchool	0.011		0.026	0.678	0.009		0.024	0.708	0.010		0.011	0.390
Education_Bachelors/Masters/PhD	0.016		0.016	0.308	0.009		0.015	0.555	-0.002		0.007	0.760
**Occupation**												
Occupation_Employed/Selfemployed			References				References				References	
Occupation_Unemployed	-0.030		0.022	0.176	-0.018		0.025	0.455	-0.007		0.011	0.555
Occupation_Homemaker	-0.035		0.023	0.123	-0.044	**	0.019	0.022	0.006		0.014	0.672
Occupation_School/Apprenticeship/University	-0.035		0.043	0.426	-0.039		0.032	0.218	-0.030	***	0.011	0.010
Occupation_Other	-0.021		0.021	0.313	-0.022		0.020	0.270	0.003		0.010	0.744
GenderFemale	-0.045	***	0.016	0.005	-0.039	**	0.016	0.013	-0.032	***	0.007	0.000
Age	0.001		0.001	0.384	0.001		0.001	0.107	-0.000		0.000	0.662
Children	0.002		0.016	0.885	0.003		0.016	0.850	0.001		0.007	0.851
Income (ln)	-0.019	*	0.011	0.100	-0.012		0.010	0.259	-0.001		0.005	0.780
Wald chi^2	765.37				789.85				2452.33			
Prob > chi^2	0.000				0.000				0.000			
Observations	2,960				2,960				10,704			

Note: * $p < 0.1$; ** $p < 0.05$; *** $p < 0.01$; For the analysis, we used a fractional multinomial logit model and report average partial effects with cluster-robust standard errors with respect to participants. The fractional multinomial logit model is estimated with the STATA 16 and the module FMLOGIT [42]

(cont'd) Table S9. Recorded Submarket Model (robustness check)

Variable	*No music consumption*											
	Balanced fractional multinomial logit (model 1)				*Weighted fractional multinomial logit (model 2)*				*Unbalanced fractional multinomial logit (model 3)*			
	APE		*SE*	*p-Value*	*APE*		*SE*	*p-Value*	*APE*		*SE*	*p-Value*
COVID-19	0.014	**	0.006	0.023	0.010	*	0.006	0.100	0.012	***	0.003	0.000
Summer	-0.004		0.005	0.358	-0.005		0.004	0.289	-0.000		0.003	0.863
Music related control variables												
MusicEducation	0.003		0.006	0.696	0.001		0.006	0.788	-0.004		0.003	0.291
MusicAppreciation	-0.011	***	0.003	0.000	-0.009	***	0.002	0.000	-0.009	***	0.001	0.000
ActiveListening	0.009	***	0.003	0.000	0.008	***	0.002	0.000	0.005	***	0.001	0.000
MainstreamMusic	-0.004		0.003	0.149	-0.004	*	0.002	0.068	-0.005	***	0.001	0.000
Demographics												
**Marital status**												
MaritalStat_LivTogether_Partner		References				References				References		
MaritalStat_LivAlone	0.027	**	0.011	0.013	0.022	**	0.009	0.012	0.024	***	0.005	0.000
MaritalStat_LivAlone_Partner	0.002		0.016	0.919	-0.003		0.012	0.765	-0.004		0.007	0.495
**Education**												
Education_MiddleSchool /Technical/HighSchool		References				References				References		
Education_NoSchool/ElementarySchool	0.019		0.015	0.205	0.016		0.013	0.223	0.005		0.006	0.464
Education_Bachelors/Masters/PhD	-0.011		0.008	0.197	-0.007		0.007	0.356	0.001		0.005	0.883
**Occupation**												
Occupation_Employed/Selfemployed		References				References				References		
Occupation_Unemployed	0.004		0.013	0.745	0.007		0.012	0.571	0.018	**	0.009	0.034
Occupation_Homemaker	0.013		0.017	0.459	0.018		0.018	0.321	0.018	*	0.010	0.074
Occupation_School/Apprenticeship/University	-0.013		0.014	0.345	-0.017	***	0.006	0.008	-0.016	***	0.005	0.004
Occupation_Other	0.026		0.018	0.148	0.035	*	0.021	0.088	0.019	**	0.008	0.016
GenderFemale	-0.001		0.009	0.951	-0.002		0.008	0.852	0.011	**	0.005	0.020
Age	-0.000		0.000	0.891	0.000		0.000	0.905	-0.000		0.000	0.337
Children	-0.002		0.010	0.832	-0.001		0.009	0.871	-0.004		0.005	0.453
Income (ln)	-0.007		0.006	0.274	-0.007		0.005	0.181	-0.007	**	0.003	0.025
Wald chi^2	765.37				789.85				2452.33			
Prob > chi^2	0.000				0.000				0.000			
Observations	2,960				2,960				10,704			

Note: * p < 0.1; **p < 0.05; ***p < 0.01; For the analysis, we used a fractional multinomial logit model and report average partial effects with cluster-robust standard errors with respect to participants. The fractional multinomial logit model is estimated with the STATA 16 and the module FMLOGIT [42]

Table S10. Total Market Model (robustness check – random effects)

Variable	*Consumer spending (ln)*				*Music consumption (ln)*			
	Coefficient		*SE*	*p-Value*	*Coefficient*		*SE*	*p-Value*
COVID-19	-0.508	***	0.050	0.000	-0.152	***	0.025	0.000
Summer	0.089	**	0.045	0.045	0.007		0.021	0.749
Music related control variables								
MusicEducation	0.222	***	0.058	0.000	0.024		0.035	0.491
MusicAppreciation	0.148	***	0.022	0.000	0.060	***	0.012	0.000
ActiveListening	0.024		0.022	0.287	-0.029	**	0.013	0.023
MainstreamMusic	0.032		0.023	0.166	-0.012		0.014	0.413
**Purchase reason**								
PurchaseReason_Atmosphere	1.741	***	0.133	0.000				
PurchaseReason_Flexibility	1.079	***	0.114	0.000				
PurchaseReason_Habit	0.687	***	0.089	0.000				
PurchaseReason_SoundQuality	1.116	***	0.116	0.000				
PurchaseReason_Mobility	1.006	***	0.182	0.000				
PurchaseReason_Other	0.192	**	0.083	0.022				
Demographics								
**Marital status**								
MaritalStat_LivTogether_Partner			References				References	
MaritalStat_LivAlone	-0.043		0.101	0.669	0.044		0.071	0.540
MaritalStat_LivAlone_Partner	0.089		0.153	0.561	-0.113		0.080	0.158
**Education**								
Education_MiddleSchool/Technical/HighSchool			References				References	
Education_NoSchool/ElementarySchool	-0.004		0.128	0.973	-0.118		0.110	0.283
Education_Bachelors/Masters/PhD	0.001		0.107	0.995	-0.084		0.055	0.125
**Occupation**								
Occupation_Employed/Selfemployed			References				References	
Occupation_Unemployed	-0.185		0.129	0.152	-0.167	**	0.085	0.049
Occupation_Homemaker	-0.082		0.148	0.579	-0.192	**	0.090	0.032
Occupation_School/Apprenticeship/University	0.100		0.250	0.690	-0.082		0.145	0.574
Occupation_Other	-0.057		0.128	0.656	-0.119		0.086	0.165
Age	-0.305	***	0.093	0.001	-0.213	***	0.070	0.002
Children	-0.007	*	0.004	0.094	-0.001		0.003	0.643
Income (ln)	0.147		0.100	0.139	0.094		0.069	0.170
Overall R^2	0.314				0.106			
Within R^2	0.160				0.024			
Observations	2,970				2,960			

Note: * $p < 0.1$; **$p < 0.05$; ***$p < 0.01$; For the analysis, we used balanced panel random effect estimations with robust standard errors in Stata 16.

Table S11. Live Market Model (robustness check – random effects)

Variable	*Consumer spending (ln)*				*Music consumption (ln)*			
	Coefficient		*SE*	*p-Value*	*Coefficient*		*SE*	*p-Value*
COVID-19	-0.619	***	0.051	0.000	-0.143	***	0.015	0.000
Summer	0.146	***	0.039	0.000	0.059	***	0.013	0.000
Music related control variables								
MusicEducation	0.164	***	0.046	0.000	0.046	**	0.018	0.010
MusicAppreciation	0.014		0.016	0.377	0.016	***	0.005	0.001
ActiveListening	0.021		0.018	0.243	0.015	**	0.006	0.022
MainstreamMusic	-0.009		0.019	0.635	-0.010	**	0.005	0.031
**Purchase reason**								
PurchaseReason_Atmosphere	1.694	***	0.142	0.000				
PurchaseReason_Flexibility	0.064		0.086	0.459				
PurchaseReason_Habit	0.257	***	0.066	0.000				
PurchaseReason_SoundQuality	0.326	***	0.088	0.000				
PurchaseReason_Mobility	0.032		0.154	0.835				
PurchaseReason_Other	0.084		0.062	0.178				
Demographics								
**Marital status**								
MaritalStat_LivTogether_Partner		References				References		
MaritalStat_LivAlone	-0.044		0.068	0.521	-0.021		0.021	0.319
MaritalStat_LivAlone_Partner	0.145		0.108	0.179	0.027		0.035	0.434
**Education**								
Education_MiddleSchool/Technical/HighSchool		References				References		
Education_NoSchool/ElementarySchool	-0.124	*	0.067	0.064	-0.026		0.029	0.376
Education_Bachelors/Masters/PhD	-0.002		0.074	0.974	0.040		0.026	0.119
**Occupation**								
Occupation_Employed/Selfemployed		References				References		
Occupation_Unemployed	-0.101		0.080	0.205	-0.026		0.022	0.228
Occupation_Homemaker	-0.013		0.108	0.900	0.022		0.039	0.581
Occupation_School/Apprenticeship/University	0.135		0.197	0.493	0.116		0.114	0.312
Occupation_Other	-0.004		0.081	0.958	-0.010		0.029	0.745
Age	0.007		0.059	0.911	-0.011		0.022	0.621
Children	-0.001		0.003	0.742	0.000		0.001	0.761
Income (ln)	0.043		0.066	0.511	0.010		0.021	0.639
Overall R^2	0.233				0.0765			
Within R^2	0.199				0.0500			
Observations	2,970				2,960			

Note: * $p < 0.1$; ** $p < 0.05$; *** $p < 0.01$; For the analysis, we used balanced panel random effect estimations with robust standard errors in Stata 16.

Table S12. Recorded Market Model (robustness check – random effects)

Variable	Consumer spending (ln)				Music consumption (ln)			
	Coefficient		*SE*	*p-Value*	*Coefficient*		*SE*	*p-Value*
COVID-19	-0.166	***	0.039	0.000	-0.131	***	0.025	0.000
Summer	-0.029		0.036	0.431	-0.002		0.021	0.926
Music related control variables								
MusicEducation	0.140	***	0.051	0.005	0.023		0.034	0.505
MusicAppreciation	0.159	***	0.019	0.000	0.059	***	0.013	0.000
ActiveListening	0.010		0.020	0.626	-0.032	**	0.012	0.011
MainstreamMusic	0.013		0.020	0.508	-0.012		0.014	0.380
**Purchase reason**								
PurchaseReason_Atmosphere	0.442	***	0.099	0.000				
PurchaseReason_Flexibility	0.940	***	0.103	0.000				
PurchaseReason_Habit	0.511	***	0.072	0.000				
PurchaseReason_SoundQuality	0.891	***	0.101	0.000				
PurchaseReason_Mobility	1.013	***	0.174	0.000				
PurchaseReason_Other	0.115	*	0.064	0.070				
Demographics								
**Marital status**								
MaritalStat_LivTogether_Partner			References				References	
MaritalStat_LivAlone	-0.022		0.096	0.821	0.050		0.072	0.481
MaritalStat_LivAlone_Partner	-0.027		0.134	0.840	-0.119		0.082	0.146
**Education**								
Education_MiddleSchool/Technical/HighSchool			References				References	
Education_NoSchool/ElementarySchool	0.030		0.117	0.796	-0.113		0.108	0.298
Education_Bachelors/Masters/PhD	0.005		0.099	0.958	-0.096	*	0.055	0.079
**Occupation**								
Occupation_Employed/Selfemployed			References				References	
Occupation_Unemployed	-0.188	*	0.114	0.098	-0.159	*	0.085	0.062
Occupation_Homemaker	-0.155		0.119	0.193	-0.203	**	0.090	0.025
Occupation_School/Apprenticeship/University	-0.046		0.171	0.787	-0.123		0.133	0.356
Occupation_Other	-0.134		0.107	0.211	-0.119		0.086	0.169
Age	-0.358	***	0.088	0.000	-0.210	***	0.071	0.003
Children	-0.006		0.004	0.133	-0.002		0.003	0.603
Income (ln)	0.116		0.093	0.211	0.098		0.069	0.157
Overall R^2	0.284				0.105			
Within R^2	0.061				0.020			
Observations	2,970				2,960			

Note: * $p < 0.1$; ** $p < 0.05$; *** $p < 0.01$; For the analysis, we used balanced panel random effect estimations with robust standard errors in Stata 16.

Table S13. Covid-19 impact on subsample level

	Consumer spending (ln)						*Music consumption (ln)*					
	Male gender	*Sig.*	*Female gender*	*Sig.*	*Differences*	*Sig.*	*Male gender*	*Sig.*	*Female gender*	*Sig.*	*Differences*	*Sig.*
Total market	-0.432	***	-0.572	***	-0.141		-0.134	***	-0.156	***	-0.022	
Live market	-0.565	***	-0.699	***	-0.134		-0.145	***	-0.144	***	0.001	
Recorded market	-0.141	**	-0.162	***	-0.021		-0.116	***	-0.133	***	-0.017	
	Young generation	*Sig.*	*Old generation*	*Sig.*	*Differences*	*Sig.*	*Young generation*	*Sig.*	*Old generation*	*Sig.*	*Differences*	*Sig.*
Total market	-0.467	***	-0.597	***	-0.129		-0.164	***	-0.122	***	0.042	
Live market	-0.628	***	-0.640	***	-0.012		-0.144	***	-0.145	***	-0.001	
Recorded market	-0.115	**	-0.260	***	-0.145		-0.141	***	-0.107	**	0.034	
	High music appreciation	*Sig.*	*Low music appreciation*	*Sig.*	*Differences*	*Sig.*	*High music appreciation*	*Sig.*	*Low music appreciation*	*Sig.*	*Differences*	*Sig.*
Total market	-0.689	***	-0.262	***	-0.427	***	-0.119	***	-0.155	***	0.036	
Live market	-0.787	***	-0.426	***	-0.360	***	-0.154	***	-0.113	***	-0.042	
Recorded market	-0.283	***	0.016		-0.300	***	-0.098	**	-0.139	***	0.041	
	High music education	*Sig.*	*Low music education*	*Sig.*	*Differences*	*Sig.*	*High music education*	*Sig.*	*Low music education*	*Sig.*	*Differences*	*Sig.*
Total market	-0.566	***	-0.454	***	-0.112		-0.173	***	-0.125	***	-0.049	
Live market	-0.885	***	-0.456	***	-0.429	***	-0.235	***	-0.081	***	-0.154	***
Recorded market	-0.147	**	-0.161	***	0.014		-0.140	***	-0.114	***	-0.026	

Note: * $p < 0.1$; ** $p < 0.05$; *** $p < 0.01$; N = 2970 observations (594 respondents) for consumer spending N = 2960 observations (592 respondents) for music consumption; For the analysis, we used balanced panel fixed effects estimation with robust standard errors in Stata 16. Young and old generations, high and low music appreciation, and high and low music education are based on a median split in the first wave. The results refer to subsample analysis of our main models including all control variables. The difference between the separate subsamples is tested by including interactions of the grouping dummy variable, with all our independent variables in our main models based on the full balanced dataset.
